# Ring-fusion as a perylenediimide dimer design concept for high-performance non-fullerene organic photovoltaic acceptors†
†Electronic supplementary information (ESI) available: Additional information describing the synthesis of the PDI dimers and more detailed fsTA and AFM data is provided. PL-quenching data and computational details are also provided. See DOI: 10.1039/c5sc04956c


**DOI:** 10.1039/c5sc04956c

**Published:** 2016-02-09

**Authors:** Patrick E. Hartnett, H. S. S. Ramakrishna Matte, Nicholas D. Eastham, Nicholas E. Jackson, Yilei Wu, Lin X. Chen, Mark A. Ratner, Robert P. H. Chang, Mark C. Hersam, Michael R. Wasielewski, Tobin J. Marks

**Affiliations:** a Department of Chemistry and the Materials Research Center , The Argonne-Northwestern Solar Energy Research Center , Northwestern University , 2145 Sheridan Road , Evanston , Illinois 60208 , USA . Email: m-wasielewski@northwestern.edu ; Email: t-marks@northwestern.edu; b Department of Materials Science and Engineering and the Materials Research Center , The Argonne-Northwestern Solar Energy Research Center , Northwestern University , 2145 Sheridan Road , Evanston , Illinois 60208 , USA

## Abstract

A series of perylenediimide (PDI) dimers are evaluated as acceptors for organic photovoltaic (OPV) cells. The materials are characterized using a wide variety of physical and computational techniques. These dimers are first linked at the bay position of each PDI molecule *via* an aromatic spacer; subsequent photocyclization affords ring-fused dimers. Thus, photocyclization of the thiophene-linked dimer 2,5-bis-[*N*,*N*′-bis-perylenediimide-1-yl]-thiophene (**T1**) affords the twisted acceptor [2,3-*b*:2′,3′-*d*]-bis-[*N*,*N*′-bis-perylenediimide-1,12-yl]-thiophene (**T2**), while photocyclization of the thienothiophene-linked dimer, 2,5-bis-[*N*,*N*′-bis-perylenediimide-1-yl]-thienothiophene (**TT1**) affords the planar acceptor [2,3-*b*:2′,3′-*d*]-bis-[*N*,*N*′-bis-perylenediimide-1,12-yl]-thienothiophene (**TT2**). Furthermore, a dimer linked by a phenylene group, 1,4-bis-[*N*,*N*′-bis-perylenediimide-1-yl]-benzene (**Ph1**), can be selectively photocyclized to form either the twisted dimer, [1,2:3,4]-bis-[*N*,*N*′-bis-perylenediimide-1,12-yl]-benzene (**Ph1a**) or the planar dimer [1,2:4,5]-bis-[*N*,*N*′-bis-perylenediimide-1,12-yl]-benzene (**Ph2b**). Ring-fusion results in increased electronic coupling between the PDI units, and increased space-charge limited thin film electron mobility. While charge transport is efficient in bulk-heterojunction blends of each dimer with the polymeric donor **PBDTT-FTTE**, in the case of the twisted dimers ring fusion leads to a significant decrease in geminate recombination, hence increased OPV photocurrent density and power conversion efficiency. This effect is not observed in planar dimers where ring fusion leads to increased crystallinity and excimer formation, decreased photocurrent density, and decreased power conversion efficiency. These results argue that ring fusion is an effective approach to increasing OPV bulk-heterojunction charge carrier generation efficiency in PDI dimers as long as they remain relatively amorphous, thereby suppressing excimer formation and coulombically trapped charge transfer states.

## Abstract 2

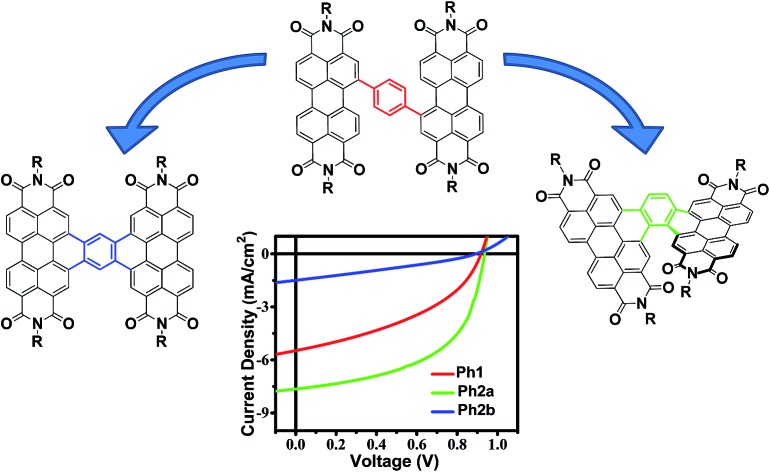
A series of perylenediimide (PDI) dimers are evaluated as acceptors for organic photovoltaic (OPV) cells.

## Introduction

Organic photovoltaic (OPV) devices offer a potential avenue for inexpensive, mechanically flexible, and environmentally friendly solar energy conversion.^1^–^10^ The most efficient OPV active layers are based on blending an electron donating material with an electron accepting material to form a phase-separated bulk-heterojunction (BHJ) structure, although both bilayer and single component active layer organic devices have also been demonstrated.^11^–^16^ By far the most heavily used acceptor materials in BHJ devices are fullerene derivatives due to their exceptional charge transport properties. Devices based on fullerenes have achieved internal quantum efficiencies near 100%, and fill factors near 80%.^12^,^17^,^18^ Fullerenes, however, are expensive to synthesize, difficult to modify, and air-sensitive, limiting their large-scale applicability/manufacturability.^19^,^20^ Because of these challenges, much recent OPV research has shifted towards developing efficient non-fullerene acceptors.^21^–^31^ One of the most successful and widespread classes of non-fullerene acceptors are derivatives of perylenediimide (PDIs). PDIs are attractive alternatives to fullerenes since they have electron affinities comparable to those of fullerenes, can have high n-type mobilities, are inexpensive to synthesize, strongly absorb visible light, and are straightforward to chemically modify, affording diverse electronic properties and solid-state morphologies.^32^–^37^

The seemingly ideal properties of PDIs described above have stimulated implementation in OPVs for decades. In fact, the first reported bilayer OPV was fabricated using a PDI derivative as the acceptor material.^16^ However, until recently, PDI derivatives have performed poorly in OPV devices due to their tendency to crystallize in large domains and thus form excimers prior to charge separation.^38^,^39^ The principal strategy for overcoming these difficulties has been to design PDI derivatives that are predominantly amorphous in the solid state, with the exception of a recent report from this laboratory.^40^ Introducing amorphous characteristics can be accomplished by disrupting the tendency of PDI to π-stack either by utilizing bulky substituents or twisting the structures of PDI dimers from planarity.^27^,^41^–^44^ Twisted PDI dimers have been the most successful strategy to date, achieving power conversion efficiencies (PCE) above 8%.^24^,^29^–^31^,^45^–^50^

Although the efficiencies of OPVs based on twisted PDI dimers are promising and have advanced dramatically, in most cases they remain lower than those of the corresponding fullerene-based devices.^10^,^12^,^14^ One reason for this lower efficiency is that even the highest performing PDI derivatives are plagued by geminate recombination processes, a problem which is almost entirely overcome by the tendency of fullerenes to rapidly form free charge carriers.^40^,^49^,^51^ In contrast to the predictions of Onsager–Braun theory, BHJs using fullerenes as the acceptor material have been shown to undergo ultrafast charge transfer, resulting in radical pairs with no detectable coulombic binding energy.^18^,^52^–^54^ These findings have been attributed to delocalization of the anion formed after charge transfer between donor materials and fullerene clusters, leading to more rapid charge separation.^18^,^52^,^55^ This same effect, however, is not observed in PDI derivatives, leading to a measurable charge separation barrier.^56^,^57^ This difference in charge separation mechanism results from the anisotropic nature of PDI derivatives and their inability to couple to neighboring molecules in three-dimensions as efficiently as spherical fullerenes.^58^ Therefore, designing PDI based molecules capable of increased anion delocalization in the solid state is a plausible strategy for developing high-performance OPV acceptor materials.

Ring fusion in organic π-systems is an approach which has been successfully employed to increase electronic delocalization,^50^,^59^–^61^ and is particularly effective in the so-called ladder-type materials which can be either polymeric or oligomeric.^61^–^66^ Recently, helical π-extended PDI oligomers based on ring-fused ethylene bridges were synthesized and reported to exhibit remarkably high n-type mobilities and BHJ OPV power conversion efficiencies of over 8%.^48^–^50^ Ring fusion of directly linked PDI dimers, however, has been shown to decrease OPV efficiencies despite increased electron mobility,^23^ most likely reflecting loss in structural flexibility in the ring-fused systems, which in turn enhances π-stacking and crystallinity.

**Chart 1:**
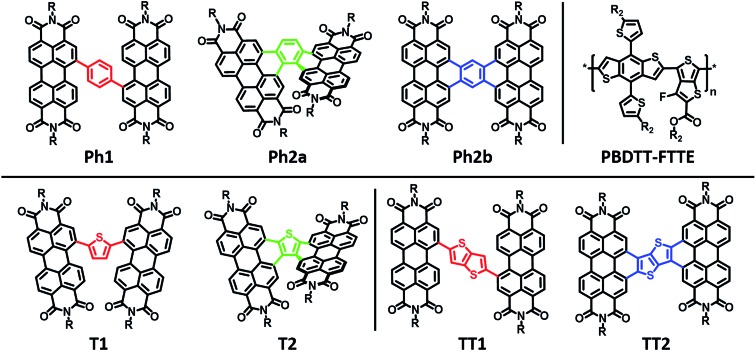
Molecular structures of the PDI acceptors and the donor polymer **PBDTT-FTTE**.

Here we explore the effects of ring fusion in a series of PDI dimers bridged by thiophene (**T1**, and **T2**), phenylene (**Ph1**, **Ph2a**, and **Ph2b**), and thienothiophene (**TT1**, and **TT2**) linkers (Chart 1) using an extensive array of experimental and computational methods.^67^ During finalization of this manuscript, Jen also compared the photophysics of **T2** and **T1**.^31^ It will be seen in the present work that steric repulsions within linked PDIs control ring-fused dimer planarity, resulting in torsion in **T2** and **Ph2a**, but allowing a high degree of planarity in both **TT2** and **Ph2b**. OPVs utilizing twisted dimers, **T2** and **Ph2a**, blended with the polymer donor **PBDTT-FTTE** as the active layer are shown here to exhibit significant increases in performance *versus* those fabricated with the analogous unfused dimers, **T1** and **Ph1**, respectively, in agreement with Jen, and together with the present results argue that rigidity of the fused molecules yields improved BHJ morphologies, decreased energetic disorder, as well as increased LUMO delocalization and electron mobility. Important support is provided by our observations of decreased performance for OPVs containing the planar dimers, **TT2** and **Ph2b**. The increased OPV performance in the twisted dimers is shown to arise from decreased geminate recombination rates as quantified here by femtosecond transient absorption spectroscopy (fsTA), to increased electron mobility, which is attributed to increased anion delocalization, as supported by electron paramagnetic resonance (EPR), and to increased connectivity as derived from Kirchoff transport indices.^58^ The decreased OPV performance observed in planar dimers, **Ph2b** and **TT2**, is attributed to the increased crystallinity of these materials, as quantified by GIWAXS, which leads to increased charge recombination and excimer formation. Taken together, these results demonstrate that ring fusion is a promising design principle for dimeric PDI BHJ acceptor materials so long as π-stacking is sufficiently disrupted to avoid excessive crystallinity.

## Experimental methods

### Materials synthesis

The synthesis, purification, and characterization of the PDI materials is described in the ESI.† **PBDTT-FTTE** was purchased from Solarmer (El Monte, CA) and used as received.

### Electrochemical characterization

Cyclic voltammetry and differential pulse voltammetry were performed using a CH Instruments Model 622 electrochemical workstation on 1.0 mM solutions of all PDI acceptor materials in anhydrous CH_2_Cl_2_ with 100 mM Bu_4_N^+^ PF_6_^–^ as the supporting electrolyte. Measurements used a platinum disc working electrode, platinum wire counter electrode, and silver wire quasi-reference electrode. Potentials are referenced to ferrocene/ferrocenium as an internal standard and are reported *vs.* SCE.

### Absorption and fluorescence spectroscopy

Steady-state optical absorption spectra were measured using a Shimadzu UV-1800 spectrometer. Photoluminescence spectra were measured in right angle mode with a HORIBA Nanolog spectrofluorimeter, and PL intensities were corrected for the absorbance at the excitation wavelength.

### Photovoltaic device fabrication and characterization

OPV cells were fabricated with an inverted device structure, ITO/ZnO/active layer/MoO_3_/Ag, according to published literature procedures.^12^ The active layers were spin-coated from solutions of 1 : 2.25 (w/w) donor : acceptor in chloroform with a polymer concentration of 10 mg mL^–1^. To ensure complete polymer dissolution, active solutions were heated at 70 °C for 12 h prior to spin coating at 6000 rpm in a glovebox. DIO (1,8-diiodooctane) was added to the solutions approximately 1 h before spin coating. Photovoltaic data acquisition and refinement used the instrumentation and procedures described previously.^40^

### Space charge limited current (SCLC) mobility measurements

Space charge limited current (SCLC) mobility measurements were performed on an Agilent Technologies B1500A Semiconductor Device Analyzer. Single carrier n-type devices were fabricated on ITO coated glass with a ZnO bottom interfacial layer and a Ca/Al top interfacial layer and electrode. The semiconducting layer was a 1 : 2.25 **PBDTT-FTTE:PDI** blend with 1% DIO added. Device areas were patterned to 200 × 200 μm^2^. The current density (*J*) was measured as a function of the applied electric field (*E*), which was corrected for any built in bias and for sheet resistance of ITO (30 Ω) prior to fitting. The space charge limited regime data were fit to eqn (1), where *ε*_s_ and *L* are the1
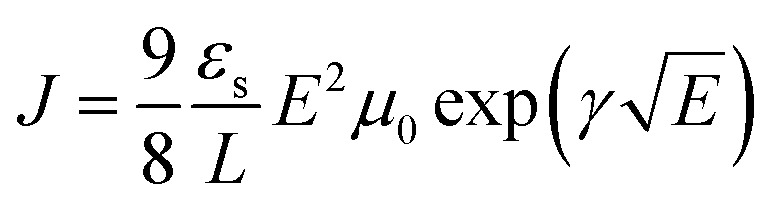
semiconductor permittivity, taken to be 3*ε*_0_, and the film thickness, respectively.^68^ The ohmic regime data were fit with a low-field carrier density model using eqn (2).

2 *J* = *η*_0_*eμ*_0_*E*

### EPR and chemical reduction

Cobaltocene (CoCp_2_), was purchased from Sigma-Aldrich and stored in an N_2_ glovebox. Under these conditions cobaltocene was found to be stable for several months with purity higher than 99.9%, as assayed in dry solvent by oxidation with a solution of Bu_4_N^+^ I^–^, by titrating the excess of iodine with aqueous thiosulfate (revealing negligible CoCp_2_^+^). All experiments were performed in anhydrous DMF solutions, previously degassed, in a glovebox under an N_2_ atmosphere. In all of the chemical reduction experiments, UV/vis and EPR spectra were measured on the same solutions. UV/vis absorbance spectra were recorded in a quartz cell with an optical path-length of 1 mm containing the solution of interest. Samples were prepared immediately prior to use and the solutions were sealed under N_2_ with Teflon stoppers. Experimental uncertainty: absorption maxima, ±1 nm.

EPR spectra were recorded using a Bruker Elexsys E580-X EPR spectrometer, equipped with a variable *Q* dielectric resonator (ER-4118X-MD5-W1). PDI samples were prepared by reduction with cobaltocene and the solution was loaded into quartz 1.4 mm tubes and sealed with a clear ridged UV-curing epoxy (IllumaBond 60-7160RCL). Data were recorded immediately after sample preparation. Solution CW-EPR spectra were collected with a 0.1 G modulation amplitude 5.12 ms time constant, and 20.48 ms conversion time, averaging 24 sweeps 20 G wide, centered around 351.06 mT.

### DFT calculations, EPR simulations, and network screening

All DFT calculations were carried out using ORCA.^58^ For every PDI acceptor molecule synthesized in this study, geometry optimization was performed at the B3LYP/DZVP level of theory using the conductor-like screening model (COSMO) to model the dielectric environment.^69^ For the COSMO calculation, a static dielectric constant of *ε* = 3 was chosen as representative of the solvation environment of each molecule. All geometries utilized propyl groups in place of the actual side-chains to reduce computational time and the required optimization space. Geometry optimizations were followed by single-point energy calculations at the TDDFT/B3LYP/DZVP/COSMO level of the theory to determine ground state orbital energies and excited state transition energies from the ground state and anion state in the linear response regime. All energies and geometries for these species can be found in the ESI.†

EPR hyperfine coupling constants for the phenyl-containing species were computed by performing geometry optimization of the anion state (charge = –1, multiplicity = 2) with an unrestricted Kohn–Sham (UKS) wavefunction at the B3LYP/EPR-II/COSMO level of theory, followed by a single point calculation of the isotropic and dipolar contributions to the hyperfine coupling tensor for each atom. All EPR calculations used tight convergence criteria (TIGHTSCF, Grid5, Finalgrid6).^70^ The results of the EPR hyperfine coupling constant calculations can be found in Table S1.†

Using a recently developed computational methodology,^58^ the bulk charge transport network properties of all PDI acceptor materials were screened, yielding calculated values of the Kirchoff transport index characterizing the strength and robustness of the charge transport networks formed by aggregates of the present PDI-acceptors. The results for these indices are provided in the ESI (Table S2†).

### Grazing incidence wide angle X-ray scattering (GIWAXS)

Grazing incidence X-ray scattering (GIWAXS) measurements were carried out at Beamline 8-ID-E of the Advanced Photon Source at Argonne National Laboratory. Correlation lengths were calculated using a modified Scherrer analysis after fitting the lamellar scattering peaks in the horizontal and vertical linecuts.^71^ Detailed experimental and data analysis procedures have been reported previously.^40^

### Femtosecond transient absorption (fsTA) spectroscopy

Low-fluence fsTA spectra were collected using 25 nJ, 100 fs pump pulses focused to a spot size of 1 mm and operating at a 100 kHz repetition rate in order to minimize excitation density, hence to avoid exciton annihilation. All samples were excited at 525 nm, and details of the transient absorption instrumentation and experimental methodology have been reported previously.^72^

## Results and discussion

### Materials synthesis

**Scheme 1:**
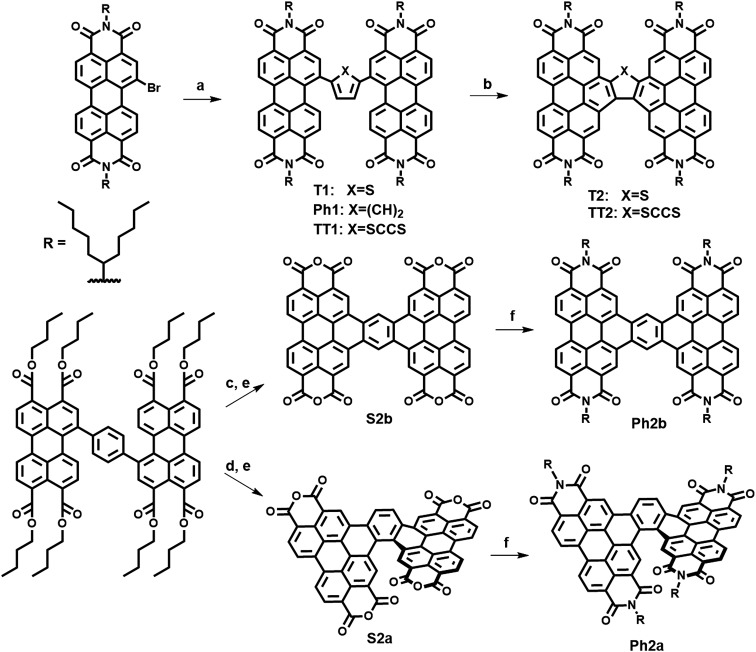
Synthesis of PDI dimers. (a) Pd(PPh_3_), toluene, (84–90%) (b) *hv*, toluene, air (84–88%) (c) CH_2_Cl_2_, I_2_, air, *hv* (d) (1) toluene, I_2_, *hv*, N_2_ (2) DDQ (e) ClSO_3_H (65–78% over 2 steps) (f) imidazole, 1-pentylhexyl.

The present new PDI acceptors were synthesized according to a common strategy (Scheme 1). In each case, the unfused precursor structure was synthesized using Pd-catalyzed cross-coupling of brominated PDI with the appropriate aromatic core to give **T1**, **TT1**, and **Ph1**. **T1** and **TT1** were then photocyclized under oxidative conditions to afford **T2** and **TT2**, respectively. In these cases, photocyclization results in one possible isomer, but in the case of **Ph1** both a linear and a twisted product are possible. Thus, pure isomers of **Ph2a** and **Ph2b** were prepared from their corresponding anhydrides, **S2a** and **S2b**, which were synthesized according to a published literature procedure where photocyclization is carried out under O_2_, to produce the twisted isomer, or N_2_ followed by oxidation with DDQ, to produce the linear isomer.^66^ Direct photocyclization of **Ph1** under conditions analogous to the photocyclization of **TT1** and **T1** was found to result predominantly in the formation of **Ph2a**, which was not isolated using this method because the use of the ester intermediate allowed for simpler purification.

### Electronic structure and transport

**Fig. 1:**
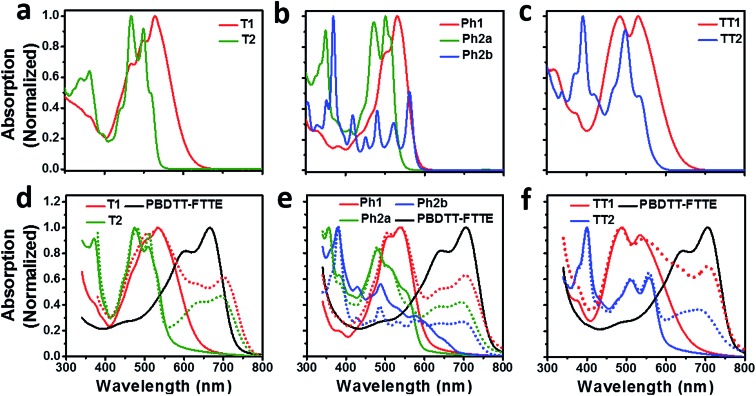
Optical absorption spectra of the PDI acceptors in solution (a–c) and as thin films, and donor polymer **PBDTT-FTTE** as a thin film (d–f solid lines). Absorption spectra of blend films cast from chloroform are indicated by the dotted lines (d–f).

Compared to an unsubstituted PDI, the solution phase optical absorption spectra of unfused structures **T1**, **TT1**, and **Ph1** are broadened and red-shifted. Upon ring fusion, the vibronic structure of the absorption sharpens and shifts to a higher energy for **T2**, **Ph2a**, and **TT2**. The absorption spectrum of **Ph2b** however shifts to lower energy and the transition is split resulting in several sharp peaks (Fig. 1a–c). The sharpening of the vibronic structure in solution is likely a result of added rigidity in the ring fused structures. When analyzed as thin films, the absorption spectra are broadened and, in the cases of **Ph2a**, **Ph2b**, and **TT2**, a new red-shifted shoulder appears (Fig. 1d–f). It is worth noting that the relative differences between the solution spectra and the film spectra are much more pronounced in the cases of **Ph2b**, and **TT2** than in the cases of **T2**, and **Ph2a**. This suggests that the intramolecular coupling is much weaker in the twisted structures than in the planar structures, consistent with previously reported data for fused PDI dimers.^31^,^49^ In **PBDTT-FTTE:PDI** blend films, the absorption of the polymer donor is observed above 600 nm while the acceptors absorb most strongly below 600 nm. This results in a broad absorption profile where the blend films absorb strongly across the entire visible spectrum. For the most part, the absorbances of the individual donor and acceptor components in the blend films are unchanged, resulting in absorption spectra which resemble the sum of the spectra of the individual components; however in the case of **T1** and **T2** the polymer absorbance is red-shifted by about 20 nm in the blend, suggesting the presence of a charge transfer (CT) interaction (Fig. 1d).

**Fig. 2:**
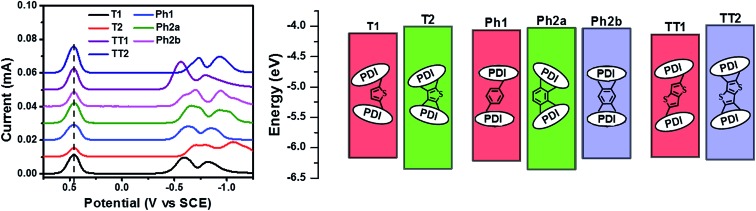
DPV profiles (left) and energy levels (right) of the present PDI acceptor molecules.

The reduction potentials of each molecule were measured electrochemically and used to estimate the LUMO levels of the acceptors (Fig. 2, Table 1). In all cases, ring fusion leads to a more negative reduction potential of between 30 mV (**Ph2b**) and 150 mV (**TT2**), consistent with increased coupling to the electron rich linker, and a larger shift in LUMO is observed for more electron rich linkers. While the first and second reductions of the unfused PDI dimers are separated by only 30–60 mV, this separation increases in the fused dimers to around 100 mV, further indicating increased coupling between the PDI molecules (Table 1).

**Table 1:** Electronic structure properties of the indicated PDI dimers

Molecule	**T1**	**T2**	**Phl**	**Ph2a**	**Ph2b**	**TT1**	**TT2**
*E*_red_ (V)	–0.56, –0.62	–0.67, –0.80	–0.61, –0.64	–0.64, –0.75	–0.59, –0.70	–0.54, –0.60	–0.69, –0.77
*E*_gap_ (eV)	2.05	2.34	2.15	2.33	2.14	2.02	2.21
HOMO (eV)	–6.17	–6.35	–6.22	–6.36	–6.18	–6.16	–6.20
LUMO (eV)	–4.12	–4.01	–4.07	–4.03	–4.04	–4.14	–3.99
*μ*_e_ (10^–5^ cm^2^ V^–l^ s^–1^)	1.2 ± 0.7	4.7 ± 0.2	1.7 ± 0.3	4.6 ± 0.8	3.2 ± 0.6	15 ± 1	48 ± 3
*K*_T_ (10^–2^)	1.0 ± 0.2	1.1 ± 0.2	1.0 ± 0.2	1.1 ± 0.2	1.2 ± 0.2	0.7 ± 0.1	1.5 ± 0.2

SCLC electron carrier mobilities were measured for films of each acceptor blended with **PBDTT-FTTE** (Table 1). In each case, ring fusion results in 3–4× increase in electron mobility, suggesting the π–π electronic connectivity of neighboring molecules increases as a result of ring fusion, enabling easier flow of charge carriers, consistent with previous reports on **T2**.^31^ The electronic connectivity of the neighboring molecules is additionally supported by our computation of the corresponding Kirchoff transport indices (*K*_T_, see ESI†). In all cases, ring fusion leads to increased connectivity with a more significant enhancement for the planar fused dimers than in the twisted dimers (Table 1). This is consistent with the measured SCLC mobilities, providing an explanation for the observed trend and suggesting that the fused structures are more capable of forming connected charge transport networks than the unfused structures.

### PDI electronic coupling

**Fig. 3:**
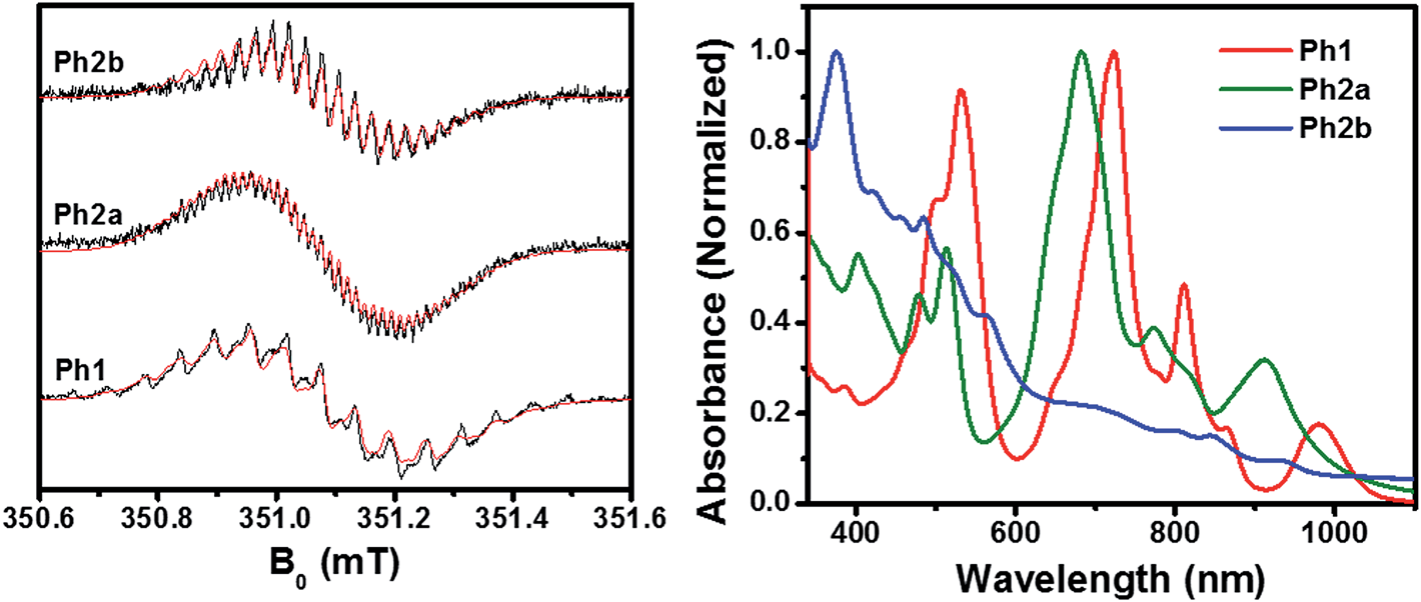
EPR (left) and UV-vis (right) spectra of chemically reduced **Ph1**, **Ph2a**, and **Ph2b** dimers in DMF solution. The spectral features of the neutral molecules are still evident since less than one equiv. of reductant was used.

As discussed above, ring fusion results in splitting of the first and second reduction potentials of the PDI dimers and increased SCLC mobility, indicating increased electronic coupling between the individual PDI cores in the fused structures. To further investigate this effect, **Ph1**, **Ph2a**, and **Ph2b** were chemically reduced using cobaltocene and the resulting radical anions were investigated using EPR and optical spectroscopy (Fig. 3). The EPR spectra of all three dimeric anions exhibit linewidths and ^14^N hyperfine coupling constants that are approximately one-half those observed for the monomeric PDI anion, suggesting that the unpaired electron is either fully delocalized or rapidly hopping between PDIs in all three dimeric systems on the timescale of the experiment (ns).^70^ The principal difference in the EPR spectra is the strong coupling between the unpaired spin and the bridge protons in the fused structures, with ^1^H hyperfine coupling constants of *a*_H_ = 1.00 MHz for **Ph2a**, and *a*_H_ = 2.44 MHz for **Ph2b**, while the unpaired spin is only weakly coupled to the bridge in **Ph1** (*a*_H_ = 0.24 MHz). These coupling constants are in agreement with computed values (Table S1†) and indicate that there is significant radical anion electron density on the bridge in the fused structures but not in the unfused structure. The electron density on the bridge in the fused structures suggests that the electron may be truly delocalized in the fused structures rather than simply hopping between the individual PDIs.

The optical absorbance spectra of the anions (Fig. 3) show that, when compared to the monomeric PDI radical anion (700, 795, and 955 nm),^73^**Ph1** is slightly red-shifted with absorption peaks at 724, 811, and 981 nm. These peak positions are similar to those observed for an anion hopping between adjacent monomeric PDIs in a solution aggregate.^70^ In the case of **Ph2a**, the anion absorption peaks are blue-shifted to 682, 773, and 910 nm and broadened relative to those of **Ph1**, while in **Ph2b** the anion features are very broad. Broadening of the anion features in **Ph2a** and **Ph2b** is suggestive of increased anion delocalization.^74^

### OPV device fabrication and evaluation

**Fig. 4:**
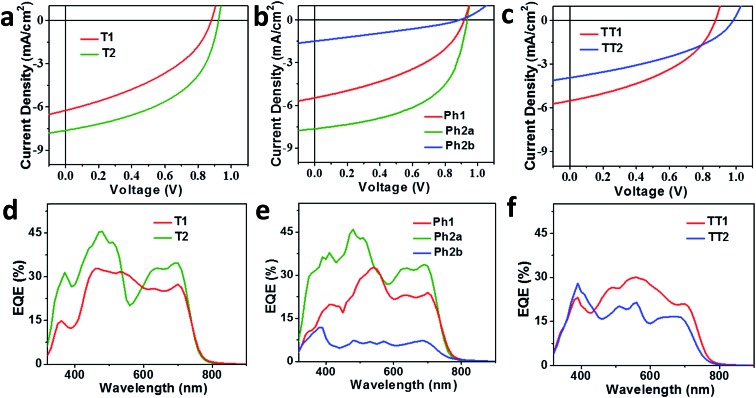
*J*–*V* curves (a–c) and EQE spectra (d–f) of OPV devices based on **T1** and **T2** (a and d), **Ph1**, **Ph2a**, and **Ph2b** (b and e), and **TT1** and **TT2** (c and f) blended with the donor polymer **PBDTT-FTTE**.

OPV devices were fabricated from each PDI acceptor using an inverted architecture (ITO/ZnO/active layer/MoO_3_/Ag) and **PBDTT-FTTE** as the donor material. The optimized device parameters are summarized in Table 2. The performance of devices based on non-planar fused structures **T2** and **Ph2a** is significantly enhanced *versus* those based on unfused structures **T1**, **TT1**, and **Ph1**, although the performance is somewhat lower than that reported for a blend of **T2** with the same polymer.^31^ This is likely due to the fact that the active layers in the devices studied were thinner leading to incomplete absorption in the film (Fig. S2†). In contrast, devices based on planar fused structures (**Ph2b** and **TT2**) have inferior performance. The *J*–*V* plots (Fig. 4a–c) show that the observed trends in PCEs reflect variations in short circuit current (*J*_SC_) and fill factor (FF). The open circuit voltages (*V*_OC_) of the materials vary predictably with variations in the acceptor LUMO levels in that there is only a slight enhancement in *V*_OC_ for the fused structures. For dimeric PDIs **T2** and **Ph2a**, an increase in both FF and *J*_SC_ is observed, while a decrease in both parameters is observed for **Ph2b** and **TT2**. The increased FF is consistent with the increased SCLC mobility of the non-planar fused structures (Table 1), however, a similar increase in SCLC mobility is also observed for **Ph2b** and **TT2** but is accompanied by decreased FF. This decrease in fill factor is likely a result of the greater crystallinity of blends having planar fused PDI dimers as will be discussed below. The changes in *J*_SC_ upon ring fusion are accompanied by parallel changes in external quantum efficiency (EQE, Fig. 4d–f). Moreover, contributions from both the donor and acceptor moieties can be seen in the EQE spectra, which closely track the absorption spectra of the blends (Fig. 1), suggesting that both donor and acceptor contribute to the photocurrent. In contrast to the non-planar dimers, there is a slight decrease in EQE for **TT2** and a large decrease for **Ph2b**. Since all of the blends absorb similarly (Fig. S1†), the observed changes in EQE and *J*_SC_ must be due to changes in the efficiency of charge separation or charge collection.

**Table 2:** OPV device performance parameters^*a*^

Molecule	**T1**	**T2**	**Ph1**	**Ph2a**	**Ph2b**	**TT1**	**TT2**
*V*_OC_ (V)	0.87	0.92	0.91	0.93	0.89	0.87	0.98
*J*_SC_ (mA cm^–2^)	6.24	7.52	5.50	7.68	1.51	5.55	3.95
FF (%)	40.1	49.5	41.3	54.3	29.3	41.7	38.9
PCE (%)	2.19	3.44	2.19	3.89	0.23	2.02	1.50

^*a*^ *JV* characteristics were measured for all devices under 100 mW cm^–2^ AM1.5G simulated solar illumination. All devices were fabricated using an inverted device architecture (ITO/ZnO/active layer/MoO_3_/Ag). Active layers were cast from 1 : 2.25 donor : acceptor chloroform solutions that contained 1% DIO by volume.

### Active layer morphology

**Fig. 5:**
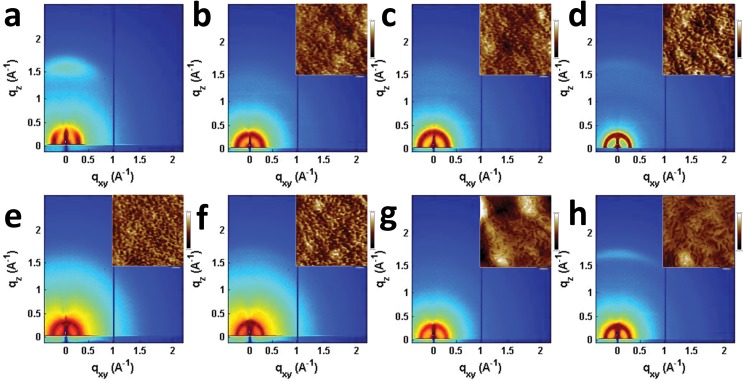
GIWAXS images of pristine **PBDTT-FTTE** (a), **PBDTT-FTTE:Ph1** (b), **PBDTT-FTTE:Ph2a** (c), **PBDTT-FTTE:Ph2b** (d), **PBDTT-FTTE:T1** (e), **PBDTT-FTTE:T2** (f), **PBDTT-FTTE:TT1** (g), and **PBDTT-FTTE:TT2** (h) films. AFM images of the blend films are shown in the insets and enlarged versions are available in Fig. S3.† All films were spin-coated on Si/SiO_2_ from chloroform with 1 wt% DIO.

Seemingly small changes in BHJ blend active layer morphology often play a significant role in OPV performance and result in large changes in device efficiency, and has been reported to play an important role in **T2** and similar systems.^31^,^46^,^75^–^78^ The morphologies of the **PBDTT-FTTE:PDI** blends discussed above were investigated using AFM and GIWAXS (Fig. 5). The AFM images of the samples show that the surface morphologies of the blend films are smooth with features in each sample being no larger than about 10 nm (Fig. 5 inset and S3†). However, GIWAXS reveals noticeable differences in the crystallinity of these materials. The pristine polymer GIWAXS scattering pattern shown in Fig. 5a suggests that the polymer sits in a face-on orientation to the substrate with a large π-stacking peak in the vertical line cut and a lamellar scattering peak in the horizontal linecut. These polymer features are minor relative to the PDI features since **PBDTT-FTTE** is a relatively amorphous polymer.^79^

**Fig. 6:**
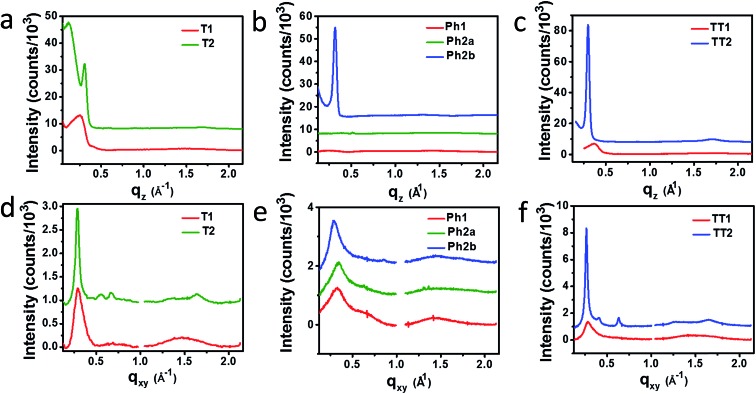
Vertical (a–c) and horizontal (d–f) GIWAXS linecuts of **PBDTT-FTTE:T1** and **PBDTT-FTTE:T2**, (a and d) **PBDTT-FTTE:Ph1**, **PBDTT-FTTE:Ph2a**, and **PBDTT-FTTE:Ph2b** (b and e), and **PBDTT-FTTE:TT1** and **PBDTT-FTTE:TT2** (c and f).

Horizontal and vertical linecuts of the GIWAXS images show that the unfused PDI dimers are relatively amorphous (Fig. 6). However, ring fusion of **T1** and **Ph1** to form **T2** and **Ph2a**, respectively, results in slightly increased crystallinity as indicated by a relative increase in scattering intensity, however the non-planar fused dimers are still more amorphous than what could be termed crystalline PDI materials.^40^ In contrast, ring fusion to form the planar fused dimers **Ph2b** and **TT2** results in a much larger increase in crystallinity, indicated by the intense and sharp scattering peaks in the linecuts. This effect can also be easily seen in the GIWAXS scattering images of the pristine acceptor films (Fig. S4†). The scattering patterns for the unfused structures have no noticeable peaks, and the scattering is mostly unchanged in **Ph2a** and **T2**, but in the case of the planar dimers, **Ph2b** and **TT2**, intense crystalline peaks are clearly visible. Scherrer analysis of the GIWAXS linecuts supports this argument showing that the average correlation length is small in **T1**, **Ph1** and **TT1**, on the order of 2–5 nm. The correlation lengths increase slightly in the twisted dimers **T2** and **Ph2a**, to 3–8 nm, but increase much more significantly in the planar dimers, ranging from 11–18 nm (Table S2†). The high degree of crystallinity observed for the planar fused dimers likely contributes to the decreased PV efficiency of devices using these materials since crystallinity in PDIs has been associated previously with excimer formation and excessive phase segregation.^80^,^81^ Additionally, there is a strong end-on orientation preference for **Ph2b**, indicated by the relative intensity of the lamellar scattering peak in the out-of-plane linecut relative to the in-plane linecut, which has been shown to be detrimental to OPV performance because of the anisotropic nature of charge transport in PDIs.^82^

### Charge separation and recombination dynamics

Photoluminescence (PL) spectra (Fig. S6†) show that there is significant excimer formation in **Ph2b** films but not in **TT2** films, despite the fact that both are crystalline materials. These results can explain why a more significant decrease in PV performance is observed for devices based on **Ph2b**. Despite this observed excimer formation, blends of all seven materials show that PL is quenched by greater than 95%, suggesting that charge transfer is nearly quantitative, although **Ph2b** shows the lowest degree of quenching, and excimer states are generally not highly emissive,^83^ so it is conceivable that charge transfer is less efficient in this case. The high yields of charge transfer suggest that the increased OPV performance upon ring fusion for the non-planar dimers and the more modest performance for the planar dimers most likely results from differences in charge recombination dynamics and possibly charge collection.

**Fig. 7:**
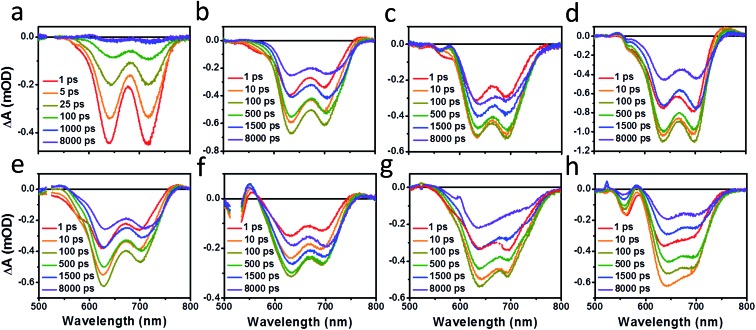
fsTA film spectra of pristine **PBDTT-FTTE** (a), **PBDTT-FTTE:Ph1** (b), **PBDTT-FTTE:Ph2a** (c), **PBDTT-FTTE:Ph2b** (d), **PBDTT-FTTE:T1** (e), **PBDTT-FTTE:T2** (f), **PBDTT-FTTE:TT1** (g), and **PBDTT-FTTE:TT2** (h).

**Fig. 8:**
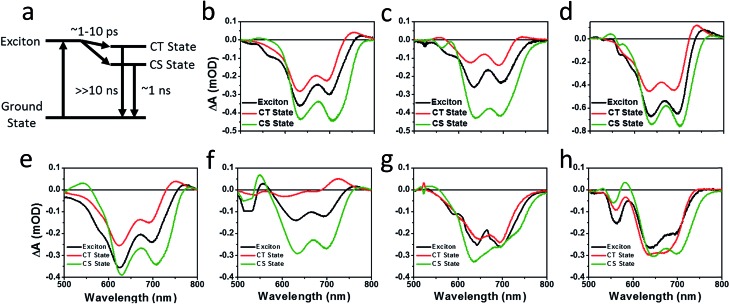
Species associated spectra obtained by fitting the fsTA spectra of **PBDTT-FTTE:Ph1** (b), **PBDTT-FTTE:Ph2a** (c), **PBDTT-FTTE:Ph2b** (d), **PBDTT-FTTE:T1** (e), **PBDTT-FTTE:T2** (f), **PBDTT-FTTE:TT1** (g), **PBDTT-FTTE:TT2** (h), to a model (a) in which the exciton forms either a trapped state (CT state), which rapidly decays to the ground state, or dissociates into free carriers (CS state), which recombine slowly.

The charge separation and recombination dynamics of the present PDI systems were further investigated using femtosecond transient absorption (fsTA) spectroscopy. The transient spectra of the blend films and of pristine **PBDTT-FTTE** are shown in Fig. 7, while transient spectra of the pristine PDI dimers can be found in the ESI (Fig. S3†). The fsTA spectral features of the blends are dominated by the polymer bleach between 550 and 750 nm resulting in the spectra of the blends looking similar with slight changes at lower wavelengths resulting from changes in the PDI absorption. The (fsTA) spectra of the blends can be fit within experimental error (Fig. S5†) to a model where an exciton state undergoes electron transfer in 1–10 ps to either a CT state, which then undergoes geminate recombination in ∼1 ns, or dissociates forming a fully charge separated (CS) state which does not recombine on the timescale of the experiment (Fig. 8a). This model must be treated as a pragmatic approximation since it does not distinguish between excitons on the PDI acceptor and excitons on the donor polymer and also does not account for energy transfer. In addition, this model does not account for the possibility of carriers in the trapped CT state dissociating into free carriers. However, the utility and value of this model can be seen from the fact that it is able to fit all seven data sets with a high degree of accuracy (Fig. S5†).

Results from fitting the fsTA spectra are shown in Fig. 8. The spectra are fits of the 2D dataset according to the model (Fig. 8a) and represent the exciton (shown in black), CT (shown in red), and CS (shown in green) states and are normalized to reflect their relative populations.

Since the polymer bleach feature dominates the spectra, the relative yields of the CT state and the CS state can be estimated by comparing their intensities between 600 and 700 nm. All three unfused systems are similar with a slightly higher yield of the CS state *versus* the CT state (Fig. 8b, e and g). In blends of the non-planar fused dimers **T2** and **Ph2**, the yields of free carriers increase dramatically and are large compared to the yield of trapped carriers, suggesting that there is little geminate recombination in these systems (Fig. 8b and f). In marked contrast, blends of the planar fused dimers exhibit increased geminate recombination relative to the non-planar fused dimers and, in the case of **TT2**, even more geminate recombination than in the unfused **TT1**, convincingly explaining why there is a decrease in PCE for BHJ films of **TT2** relative to **TT1**. These trends in geminate recombination are best explained by two mechanisms. In the fused structures, the individual PDI molecules are more tightly coupled, with the increased delocalization facilitating free carrier formation. In the case of **Ph2b** and **TT2**, this benefit is potentially offset by the fact that planar acceptors are capable of packing tightly with the polymer donor, resulting in higher coulombic binding energies and therefore a greater likelihood of forming a trapped CT state.^84^,^85^ When combined with the increased crystallinity and excimer formation in **Ph2b** and **TT2**, this increase in geminate recombination likely explains the observed trend in performance.

## Conclusions

Ring fusion is found to increase the electronic coupling between PDI acceptor molecules, resulting in increased electron mobilities and increased LUMO energies. While the increased LUMO energy leads to increased *V*_OC_s for **PBDTT-FTTE**-based BHJ OPVs based on ring-fused dimeric acceptors, the major changes in device performance reflect changes in FF and *J*_SC_. Although AFM indicates the absence of large domains in blends of any of these materials, GIWAXS shows increased crystallinity in blends of planar fused materials (**Ph2b** and **TT2**), which leads to excimer formation in the case of **Ph2b** which likely contributes to the decreased performance of devices based on these materials. GIWAXS also shows that blends of the non-planar fused dimers (**T2** and **Ph2a**) are able to remain relatively amorphous despite their increased rigidity.

PL quenching in films of all seven materials blended with **PBDTT-FTTE** shows that charge transfer is nearly quantitative, which is further confirmed by the fast rate of charge separation observed by fsTA spectroscopy of blend films. The charge carrier dynamics fit well to a model where either a trapped CT state or a completely dissociated CS state is formed upon charge separation. Ring fusion results in a significant decrease in geminate recombination in **T2** and **Ph2a** leading to improved PCEs of 3.4% and 3.9% for **T2** and **Ph2a**, respectively. Very little change in geminate recombination is observed in the case of **Ph2b** while **TT2** demonstrated an increase in recombination when compared to the unfused structures. These results are consistent with recent reports suggesting that ring fusion is capable of leading to increased anion delocalization and electron mobility.^30^,^48^ Noticeably, these properties only lead to improved performance in OPVs when the acceptor molecules are twisted, resulting in amorphous films and preventing the formation of excimers and trap states.

## Conflict of interest

The authors declare no competing financial interest.

## Supplementary Material

Supplementary information
