# Supplementary material for: Ring-fusion as a perylenediimide dimer design concept for high-performance non-fullerene organic photovoltaic acceptors
Source: Chem Sci. 2016 Feb 9;7(6):3543–55. doi: 10.1039/c5sc04956c (PMC6007210; doi:10.1039/c5sc04956c)
Supplement: Supplementary file 1 [file SC-007-C5SC04956C-s001.pdf]

Electronic Supplementary Material (ESI) for Chemical Science.
This journal is © The Royal Society of Chemistry 2016

Electronic Supplementary Information for

Ring-Fusion as a Perylenediimide Dimer Design Concept for High-Performance Non-Fullerene Organic Photovoltaic Acceptors

Patrick E. Hartnett[1], H. S. S. Ramakrishna Matte[1,2], Nicholas D. Eastham[1], Nicholas E. Jackson[1], Yilei Wu[1], Lin X. Chen[1], Mark A. Ratner[1], Robert P. H. Chang[2], Mark C. Hersam[1,2], Michael R. Wasielewski[1]* and Tobin J. Marks[1,2]*

[1] Department of Chemistry and the Materials Research Center, the Argonne-Northwestern Solar Energy Research Center, Northwestern University, 2145 Sheridan Road, Evanston, Illinois 60208, USA

[2] Department of Materials Science and Engineering and the Materials Research Center, the Argonne-Northwestern Solar Energy Research Center, Northwestern University, 2145 Sheridan Road, Evanston, Illinois, 60208, USA

Synthesis

Ring fused anhydrides **S2a** and **S2b**, and 6-bromo-N,N'-1-pentylhexyl-perylenediimide (Scheme S1) were synthesized according to published literature procedures.(REF) Solvents and reagents were purchased from Sigma-Aldrich (St. Louis, MO). $Pd(PPh_3)_4$ was purchased from TCI (Portland, Oregon). Column chromatography was performed using silica gel from Sorbent Technologies (Atlanta, GA). All solvents were spectrophotometric grade unless otherwise noted. ^{1}H nuclear magnetic resonance spectra were obtained on a Bruker Avance III 500MHz spectrometer. Matrix-assisted laser-desorption-ionization time-of-flight (MALDI-TOF) mass spectra were obtained using a Bruker Daltonics Autoflex III Smartbeam MALDI mass spectrometer in negative ionization mode without matrix.

Synthesis of T1. 6-bromo-N,N'-1-pentylhexyl-perylenediimide (500 mg, 0.64 mmol) and 2,5-bis(trimethylstannyl)thiophene (125 mg, 0.3 mmol) were dissolved in 25 mL of dry toluene and the mixture was bubbled with nitrogen. Tetrakis(triphenylphosphine)palladium(0) (35 mg, 0.03 mmol) was added and the reaction was heated to reflux for 12 hours. The mixture was cooled, dissolved in CH_2Cl_2 (100 mL), washed twice with water, dried over Na_2SO_4, filtered, and dried under reduced pressure. The crude residue was purified by silica column chromatography (3:2 CH_2Cl_2:Hexanes) to give the procuct as a dark purple powder (378 mg, 84%). ^{1}H NMR (500 MHz, Chloroform-*d*) δ 8.81 – 8.49 (m, 12H), 8.36 (m, 2H), 7.43 (br s, 2H), 5.25 – 5.16 (m, 2H), 5.12 (br s, 2H), 2.28 (m, 4H), 2.17 (br s, 4H), 1.92 – 1.76 (m, 8H), 1.39 – 1.14 (m, 48H), 0.85 (t, J = 6.9 Hz, 12H), 0.79 (t, J = 6.8 Hz, 12H). ^{13}C NMR (126 MHz, CDCl3) δ 146.72, 132.60, 129.79, 129.16, 128.82, 128.16, 127.50, 123.85, 122.95, 54.92, 32.36, 32.29, 31.78, 31.73, 26.65, 26.61, 22.59, 22.53, 14.09, 14.03. MALDI-TOF (*m/z*) $[M]^-$ = 1476.551 (Calcd for $C_{96}H_{108}N_4O_8S$: 1476.789).

Synthesis of T2. **T1** (250 mg, 0.17 mmol) and I_2 (85 mg, 0.3 mmol) were dissolved in 100 mL of toluene. The solution was bubbled with air and irradiated with a 500 W halogen lamp for 24 hours. The resulting orange solution was dried under reduced pressure and purified by silica column chromatography ($CHCl_3$) to give the product as an orange solid (220 mg, 88%). ^1H NMR (500 MHz, Chloroform-d) δ 10.60 (br s, 2H), 9.92 (br s, 2H), 9.44 (dd, J = 8.4, 5.7 Hz, 4H), 9.14 (d, J = 8.9 Hz, 4pH), 5.45-5.25 (m, 4H), 2.49 (m, 8H), 2.09 – 1.82 (m, 8H), 1.52 – 1.08 (m, 48H), 0.91 (m, 24H). ^{13}C NMR (126 MHz, $CDCl_3$) δ 167.76, 164.79, 164.18, 163.57, 140.72, 133.69, 133.33, 133.28, 132.19, 131.43, 130.88, 129.03, 128.98, 128.80, 127.73, 127.27, 125.75, 124.67, 124.63, 124.21, 123.87, 123.84, 123.68, 123.25, 123.22, 77.27, 77.01, 76.76, 32.65, 32.50, 31.84, 31.75, 26.78, 26.61, 22.63, 22.47, 14.36, 14.10, 13.90, 10.97. MALDI-TOF (*m/z*) $[M]^-$ = 1472.427 (Calcd for $C_{96}H_{104}N_4O_8S$: 1472.757).

Synthesis of Ph1. 6-bromo-N,N'-1-pentylhexyl-perylenediimide (100 mg, 0.13 mmol), benzene-1,4-diboronic acid (10.7 mg, 0.06 mmol), and potassium carbonate (450 mg, 3.25 mmol) were dissolved in a mixture of Toluene (25 mL), ethanol (2.5 mL), and water (2.5 mL). The mixture was degassed and Tetrakis(triphenylphosphine)palladium(0) (20 mg, 0.017 mmol) was added. The reaction mixture was heated to 20°C for 12 hours and then extracted with CH_2Cl_2 (100 mL) and water (50 mL). The organic layer was dried over Na_2SO_4, filtered, and dried to give a dark purple residue. The crude product was purified by silica gel chromatography (2:1 CH_2Cl_2:Hexanes) and the second red spot was collected and dried to give the product as a purple crystalline solid (76 mg, 85%). ^1H NMR (500 MHz, Chloroform-*d*) δ 8.78 – 8.58 (m, 10H), 8.44 – 8.31 (m, 1H), 8.25 – 8.12 (m, 2H), 7.94 (s, 1H), 7.69 (s, 4H), 5.31 – 5.04 (m, 4H), 2.34 – 2.14 (m, 8H), 1.94 – 1.74 (m, 8H), 1.39 – 1.13 (m, 48H), 0.89 – 0.72 (m, 24H). ^{13}C NMR (126 MHz, $CDCl_3$) δ 164.74, 163.65, 143.13, 140.48, 134.85, 134.42, 132.69, 131.61, 130.92, 130.85,

130.72, 130.02, 130.02, 128.70, 128.16, 124.02, 123.74, 123.06, 122.88, 54.88, 54.71, 32.37, 32.31, 31.79, 31.74, 26.64, 22.60, 22.55, 14.09, 14.04. MALDI-TOF (*m/z*) [*M*]$^-$ = 1471.782 (Calcd for $C_{98}H_{110}N_4O_8$: 1471.944).

Synthesis of Ph2a. **S2a** (200 mg, 0.23 mmol) was mixed with imidazole (8g) and 1-pentylhexyl amine (1 mL) and heated to 200°C overnight. The mixture was cooled, dissolved in $CHCl_3$ (100 mL), washed with water (3 x 50 mL) and dried. The crude residue was purified by silica column chromatography (CH_2Cl_2) to give the product as an orange solid (175 mg, 52%). ^{1}H NMR (500 MHz, Chloroform-*d*) δ 10.41 – 10.33 (m, 2H), 9.69 (s, 2H), 9.62 (s, 2H), 9.40 (d, J = 8.3 Hz, 2H), 9.36 (d, J = 8.3 Hz, 2H), 9.19 – 9.04 (m, 4H), 5.38 (s, 2H), 4.98 (m, 2H), 2.39 (m, 4H), 2.14 – 1.79 (m, 8H), 1.49 – 0.49 (m, 76H). ^{13}C NMR (126 MHz, CDCl3) δ 164.30, 159.60, 134.16, 133.96, 130.09, 129.03, 127.57, 127.32, 127.22, 125.87, 125.48, 124.85, 124.68, 124.46, 124.34, 123.84, 123.52, 55.18, 50.31, 34.93, 32.51, 31.83, 31.69, 31.69, 31.59, 31.37, 26.78, 26.50, 25.52, 22.62, 22.53, 14.10, 14.02. MALDI-TOF (*m/z*) [*M*]$^-$ = 1468.008 (Calcd for $C_{98}H_{106}N_4O_8$: 1467.912).

Synthesis of Ph2b. **Ph2b** was prepared in an analogous manner to **Ph2a**. **S2b** (40 mg, 0.047 mmol) was mixed with imidazole (2g) and 1-pentylhexyl amine (0.25 mL) and heated to 200°C overnight. The mixture was cooled, dissolved in $CHCl_3$ (30 mL), washed with water (3 x 10 mL) and dried. The crude residue was purified by silica column chromatography (CH_2Cl_2) to give the product as a dark purple solid (15 mg, 21%).^{1}H NMR (500 MHz, Chloroform-*d*) δ 10.74 (m, 6H), 9.00 (m, 8H), 5.43 (m, 4H), 2.47 (m, 8H), 2.05 (m, 8H), 1.55 – 1.13 (m, 48H), 0.86 (m, 24H). MALDI-TOF (*m/z*) [*M*]$^-$ = 1468.112 (Calcd for $C_{98}H_{106}N_4O_8$: 1467.912).

Synthesis of TT1. 6-bromo-N,N'-1-pentylhexyl-perylenediimide (400 mg, 0.52 mmol) and 2,5-bis(trimethylstannyl)thieno[3,2-b]thiophene (120 mg, 0.26 mmol) were dissolved in 25 mL of dry toluene and the mixture was bubbled with nitrogen. Tetrakis(triphenylphosphine)palladium(0) (10 mg, 0.01 mmol) was added and the reaction was heated to reflux for 12 hours. The mixture was cooled, dissolved in CH_2Cl_2 (100 mL), washed twice with water, dried over Na_2SO_4, filtered, and dried under reduced pressure. The crude residue was purified by silica column chromatography (8:2 CH_2Cl_2:Hexanes) to give the procuct as a dark purple powder (350 mg, 90%). ^{1}H NMR (500 MHz, Chloroform-*d*) δ 8.82 – 8.57 (m, 10H), 8.48 – 8.21 (m, 4H), 7.55 (s, 2H), 5.26 – 5.04 (m, 4H), 2.23 (m, 8H), 1.83 (m, 8H), 1.39 – 1.12 (m, 48H), 0.81 (m, 24H). ^{13}C NMR (126 MHz, $CDCl_3$) δ 164.74, 163.53, 146.97, 141.30, 134.96, 134.16, 133.97, 133.14, 131.65, 130.90, 129.95, 129.18, 129.07, 128.09, 127.49, 123.78, 122.97, 119.77, 54.74, 32.32, 31.76, 26.63, 26.61, 22.59, 22.56, 14.08, 14.06. MALDI-TOF (*m/z*) $[M]^-$ = 1534.222 (Calcd for $C_{98}H_{108}N_4O_8S_2$: 1534.058).

Synthesis of TT2. (50 mg, 0.17 mmol) and I_2 (20 mg, 0.06 mmol) were dissolved in 40 mL of toluene. The solution was irradiated with a 500 W halogen lamp for 24 hours while being bubbled with air. The resulting solution was bright red and was dried under reduced pressure and purified by silica column chromatography ($CHCl_3$) to give the product as a dark red solid (42 mg, 84%). ^{1}H NMR (500 MHz, Chloroform-*d*) δ 10.30 (s, 2H), 9.04 – 8.64 (m, 10H), 5.51 (br s, 2H), 5.09 (br s, 2H), 2.67 (m, 4H), 2.20 (m, 12H), 1.60 (m, 48H), 0.91 (m, 24H). ^{13}C NMR (126 MHz, $CDCl_3$) δ 164.66, 163.28, 142.34, 132.13, 128.61, 126.85, 126.39, 125.22, 124.48, 123.53, 122.80, 55.59, 32.63, 32.12, 31.82, 27.05, 22.78, 14.26, 14.16. MALDI-TOF (*m/z*) $[M]^-$ = 1530.187 (Calcd for $C_{98}H_{104}N_4O_8S_2$: 1530.026).

DFT Calculations: For all PDI-acceptor species, the following computations were performed using ORCA 3.0.3.

Geometry Optimization was performed at the B3LYP/DZVP level of theory using the Conductor-like Screening Model (COSMO) to model the dielectric environment. A static dielectric constaint of epsilon = 3 was chosen as representative of the solvation environment of each molecule. All geometries substituted propyl groups in place of the actual side-chain to reduce computational time and reduce the optimization space. Geometry optimizations were following by single-point energy calculations at the TDDFT/B3LYP/DZVP/COSMO level of the theory to determine ground state orbital energies and excited state transition energies in the linear response regime. Good agreement was found between computed excited state energies and the ground state absorption spectrum of each species. All values are reported in the SI.

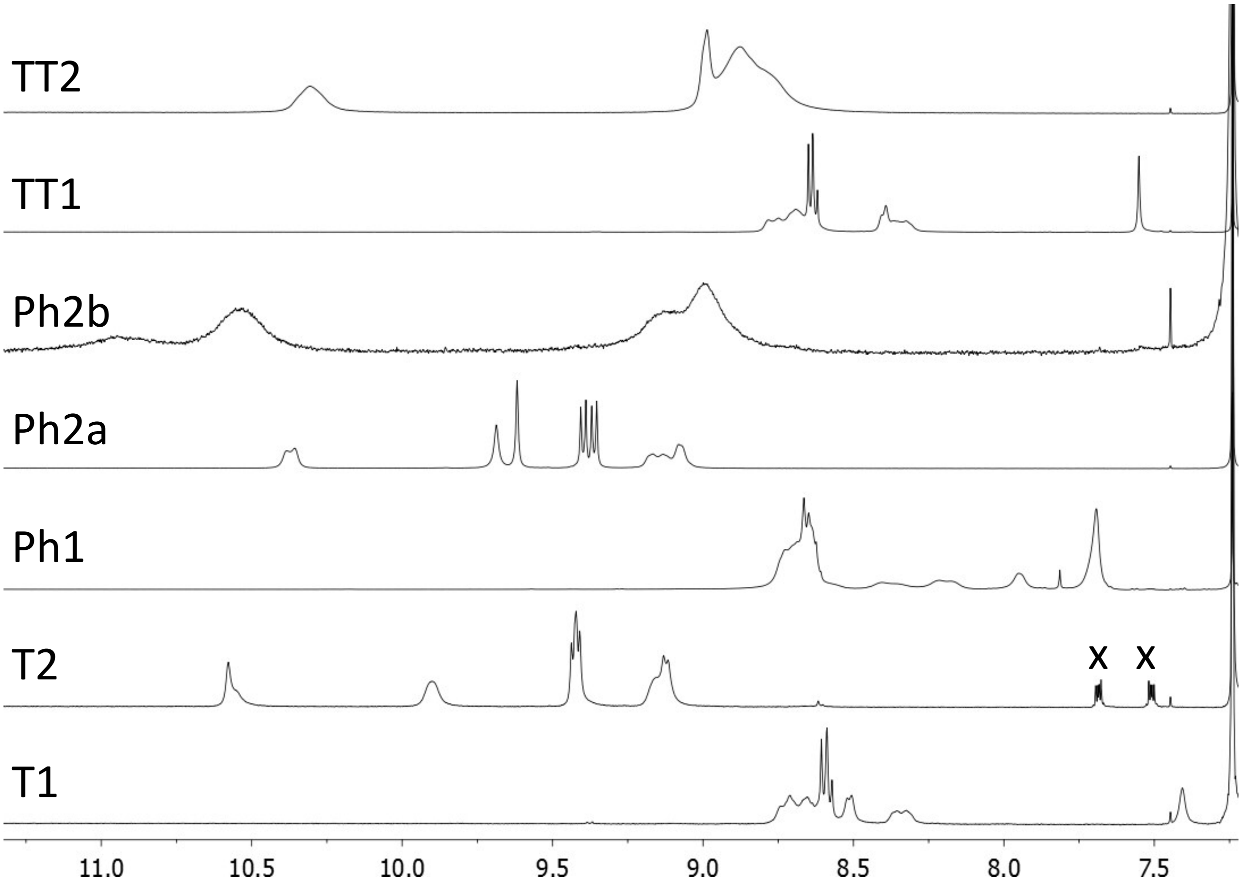

Figure S1. Aromatic region of ^{1}H NMR spectra of the PDI acceptors.

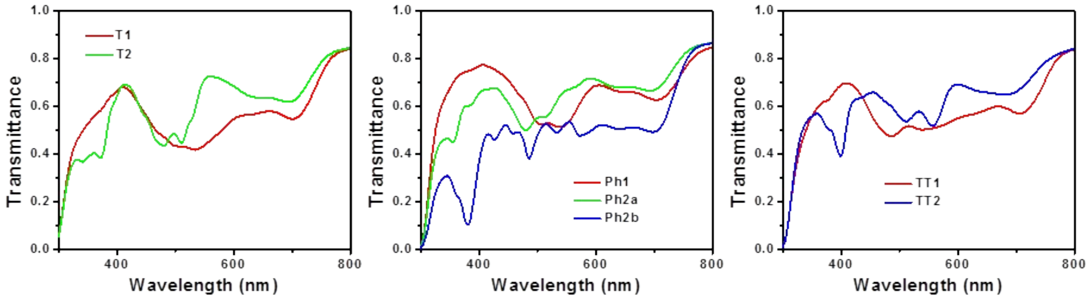

Figure S2. Transmittance spectra of blend films of the same thickness as device active layers.

Table S1. EPR hyperfine coupling constants (MHz).

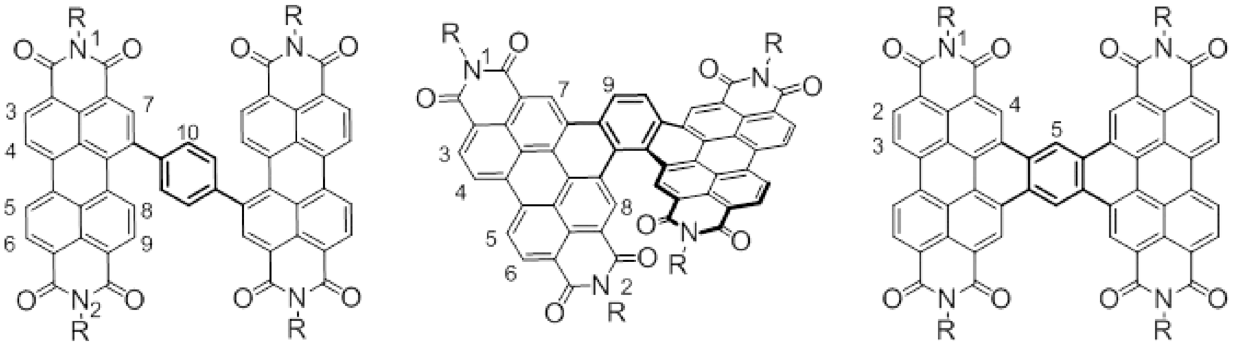

	a_{N1}	a_{N2}	a_{H1}	a_{H2}	a_{H3}	a_{H4}	a_{H5}	a_{H6}	a_{H7}	a_{H8}
Ph1	0.721 (0.741)	0.855 (0.842)	1.295 (1.344)	1.675 (1.783)	2.981 (3.043)	0.220 (0.180)	0.018 (0.031)	3.187 (3.451)	0.223 (0.291)	0.242 (0.250)
Ph2a	0.897* (0.991)	0.897* (0.795)	0.495 (0.560)	3.038 (3.384)	1.597 (1.782)	1.065 (1.186)	1.982 (2.206)	1.225 (1.350)	1.001 (1.123)	-
Ph2b	0.802 (0.801)	-	0.924 (0.928)	2.311 (2.421)	0.076 (0.004)	2.401 (2.418)	-	-	-	-

Experimental values were obtained from fitting with EasySpin[1] and calculated values are given in parenthesis. *Nitrogen hyperfine coupling constants in **Ph2a** were set to be equal to facilitate convergence of the fit.

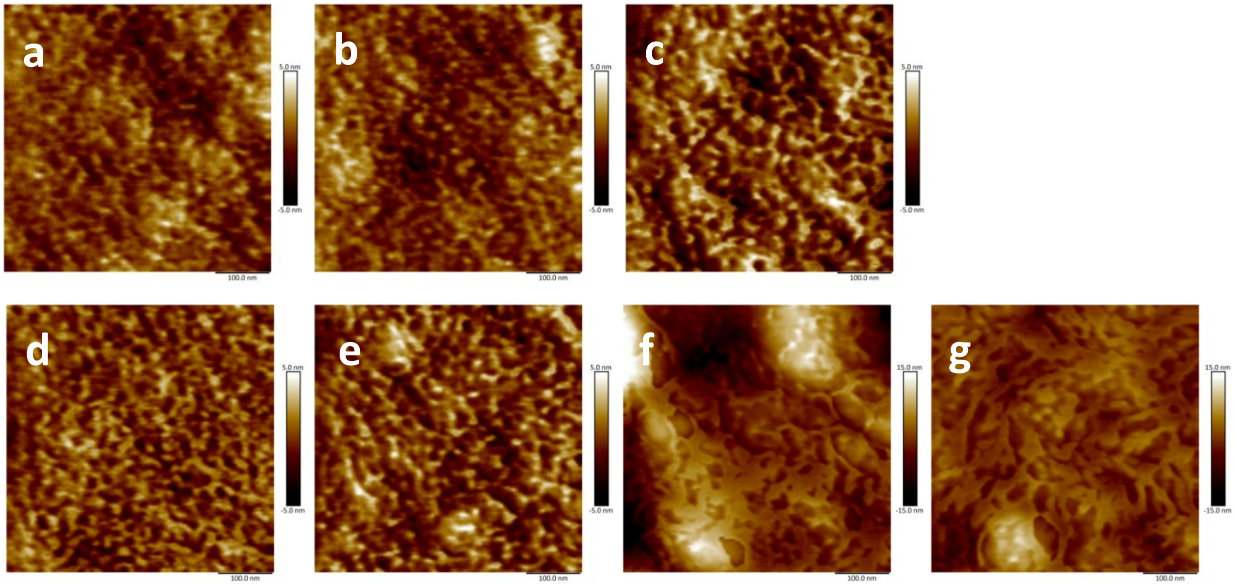

Figure S3: AFM images of **PBDTT-FTTE**:**Ph1** (a), **PBDTT-FTTE**:**Ph2a** (b), **PBDTT-FTTE**:**Ph2b** (c), **PBDTT-FTTE:T1** (d), **PBDTT-FTTE**:**T2** (e), **PBDTT-FTTE**:**TT1** (f), and **PBDTT-FTTE**:**TT2** (g)**.** The images are 1μm^2 and the height scale goes from -5 nm to 5 nm for a-e and -15 nm to 15 nm for f-g.

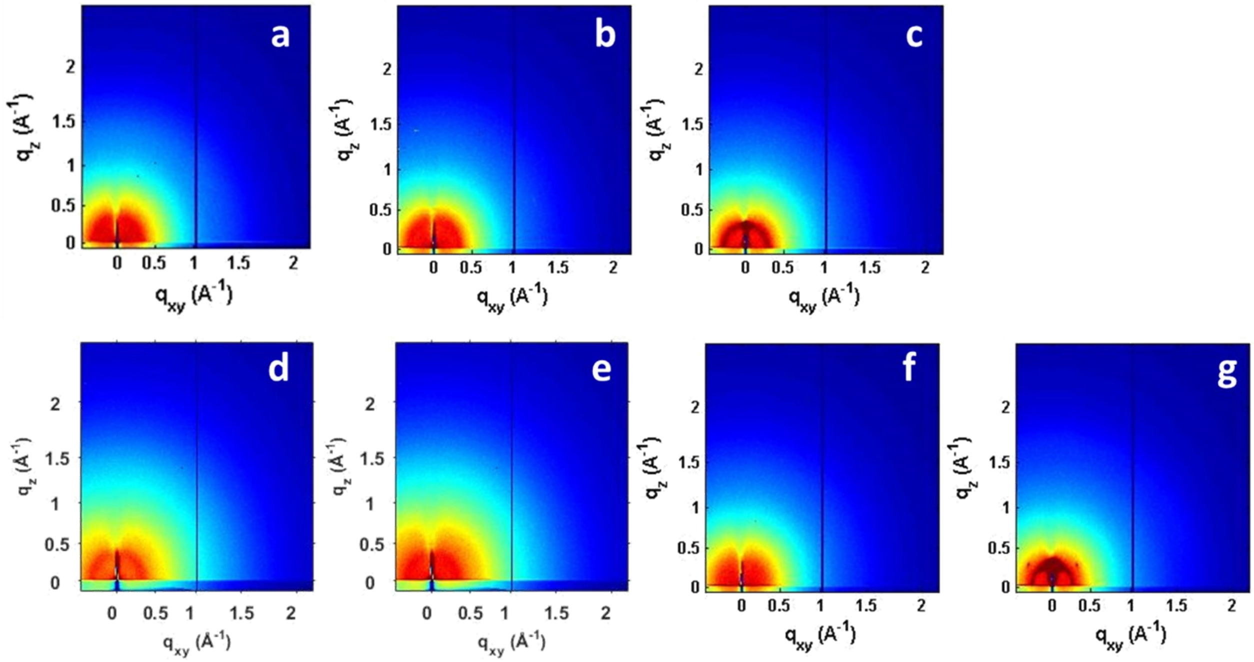

Figure S4: GIWAXS images of pristine films of **Ph1** (a), **Ph2a** (b), **Ph2b** (c), **T1** (d), **T2** (e), **TT1** (f), and **TT2** (g)**.** The images are 1μm^2 and the height scale goes from -5 nm to 5 nm for a-e and -15 nm to 15 nm for f-g.

Table S2. Correlation lengths obtained by Scherrer analysis of GIWAXS.

Acceptor	Horizontal Lamellar (Å^{-1})	D_{hkl} (nm)	Vertical Lamellar (Å^{-1})	D_{hkl} (nm)
**T1**	0.301	4.70	0.277	6.84
**T2**	0.285	6.67	0.304	8.59
**TT1**	0.292	4.96	0.267	5.20
**TT2**	0.265	18.2	0.299	14.7
**Ph1**	0.314	1.82	0.267	2.63
**Ph2a**	0.329	2.82	0.387	4.48
**Ph2b**	0.296	4.26	0.316	11.7

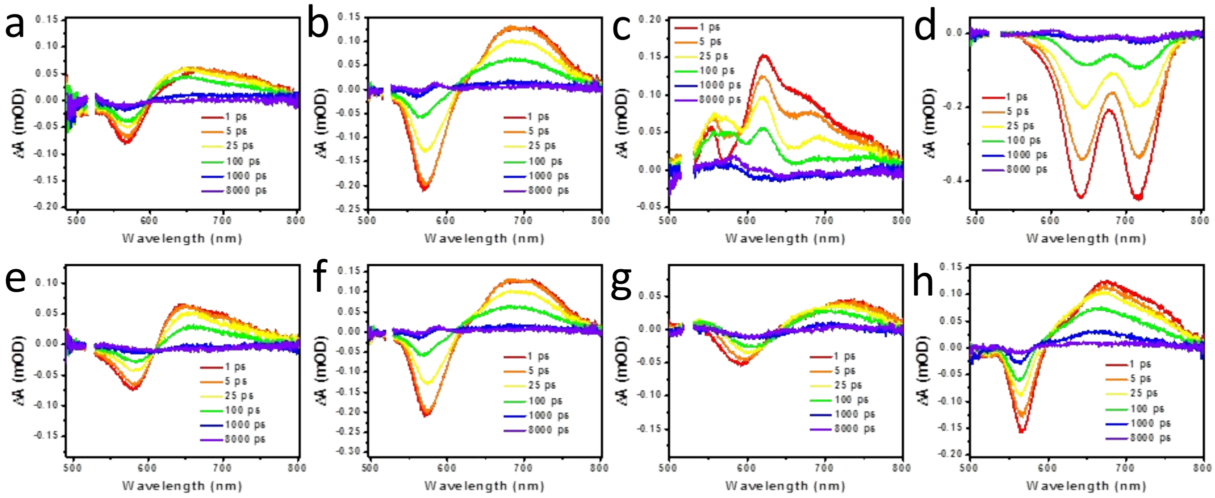

Figure S5: fsTA spectra of pristine films of **Ph1** (a), **Ph2a** (b), **Ph2b** (c), **PBDTT-FTTE** (d), **T1** (e), **T2** (f),**TT1** (g), and **TT2** (h)**.**

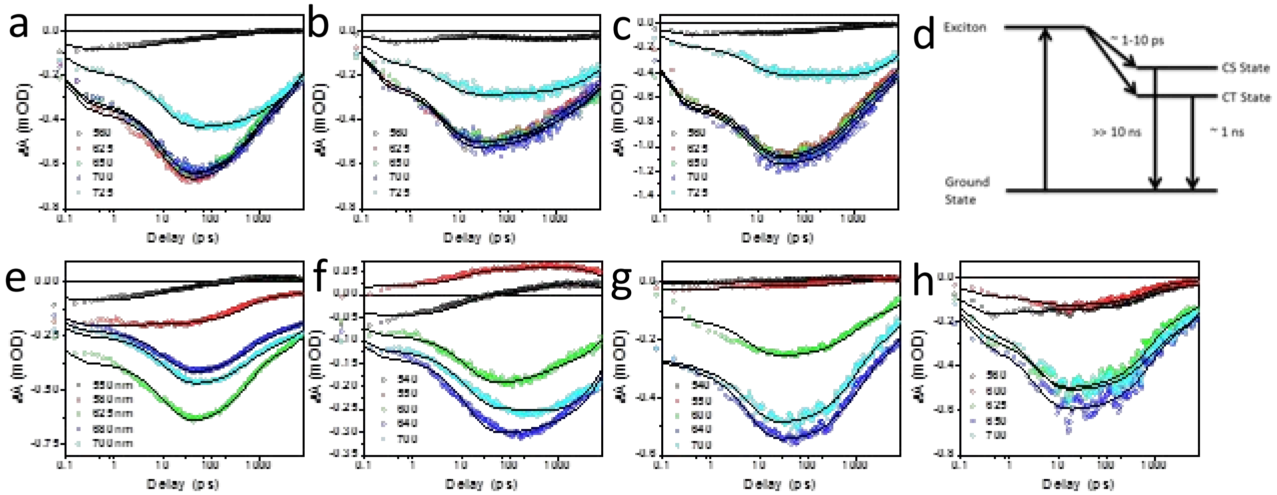

Figure S6: Kinetic fits of fsTA spectra of **PBDTT-FTTE:Ph1** (a), **PBDTT-FTTE:Ph2a** (b), **PBDTT-FTTE:Ph2b** (c), **PBDTT-FTTE:T1** (e), **PBDTT-FTTE:T2** (f), **PBDTT-FTTE:TT1** (g), and **PBDTT-FTTE:TT2** (h) fit to the model given (d)**.**

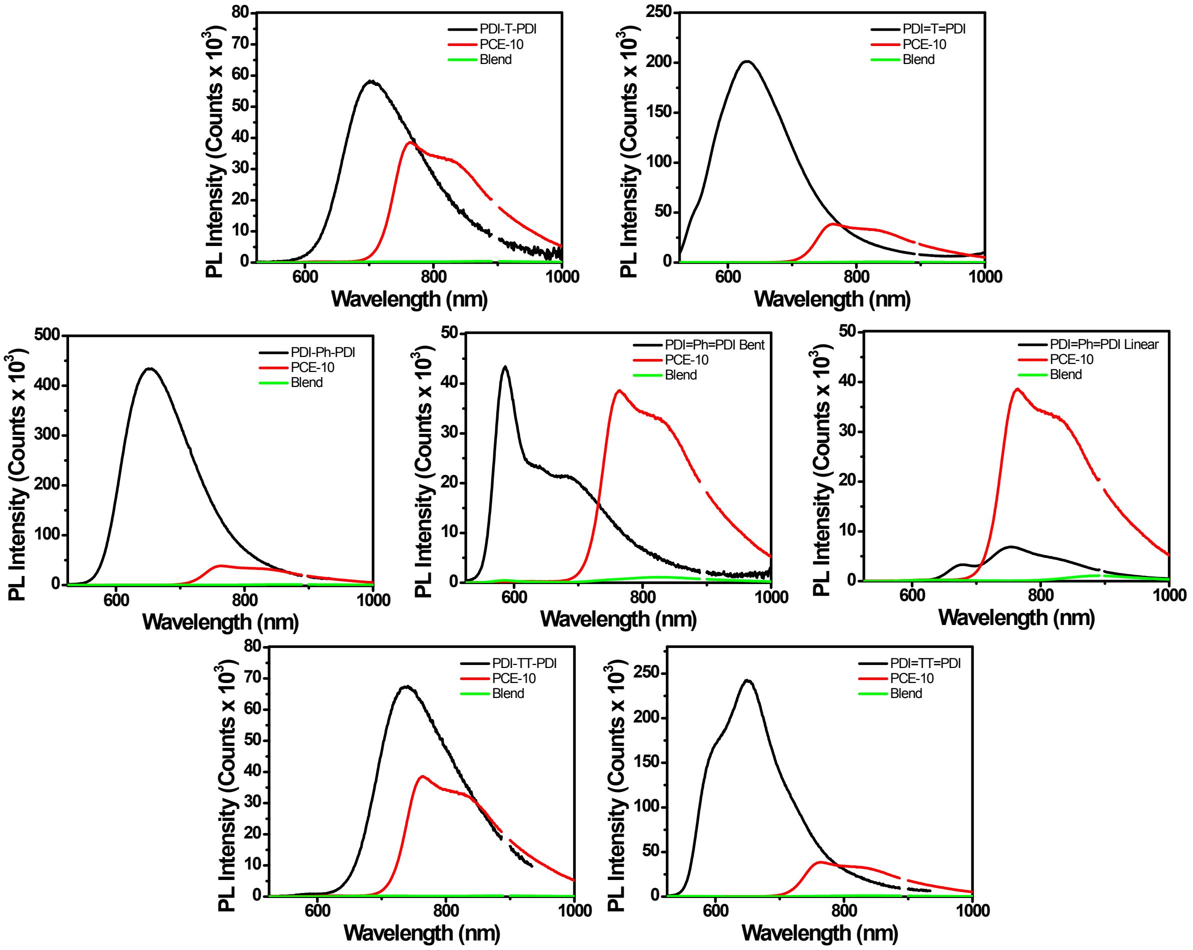

Figure S7: Photoluminescence spectra of pristine and blend films.

Calculation of the Kirchoff Transport Index

The Kirchoff Transport Index (K_T) is a qualitative characterization of the network coupling topology composed of an aggregate of many of a particular molecule. Since it does not explicitly take into account terms like the free-energy difference, ΔG, or the reorganization energy, λ, it cannot be used to quantitatively correlate mobilities across chemical species with distinct molecular structure differences. However, amongst families of chemical structures, it should qualitatively reproduce trends in mobility and charge diffusion, as has been shown in our previous work. For all species below, it is shown that K_T increases noticeably following ring

fusion, in qualitative agreement with SCLC electorn mobilities. Additionally, the characterization of spatial connectivity amongst all species follows the trend for K_T that unfused < fused bent < fused linear., which supports our hypothesis about the advantages of fused PDI structures over unfused PDI structures in forming connected charge transport networks.

Table S3. Kirchoff transport index and SCLC mobilities of the PDI dimers.

Species	Kirchoff Transport Index	SCLC Mobility (10^{-5})
**Ph1**	.00952 +/- .00157	1.7 +/- 0.3
**Ph2a**	.01084 +/- .00162	4.6 +/- 0.8
**Ph2b**	.01227 +/- .00153	3.2 +/- 0.6
**T1**	.00974 +/- .00155	1.2 +/- 0.7
**T2**	.01095 +/- .00170	4.7 +/- 0.2
**TT1**	.00737 +/- .00133	15 +/- 1
**TT2**	.01537 +/- .00215	48 +/- 3

DFT Calculations

Excited State Character of Ground State Absorption

The TDDFT ground state absorptions shown in Table S4 reproduce the experimentally observed visible absorption spectra for our PDI species. By observing the orbital character of the lowest lying excited state transitions (example for TT1 provided: transition is predominantly HOMO -> LUMO in character) it becomes apparent that the excitation possesses significant charge transfer character (Figure S7), which is correlated with the low-energy nature of the transition. By substituting the molecular structure of the connecting unit, one can provide an additional fine-tuning knob for manipulating the fused PDI structures to better absorb the solar spectrum, all through the manipulation of the charge-transfer character of the excitation.

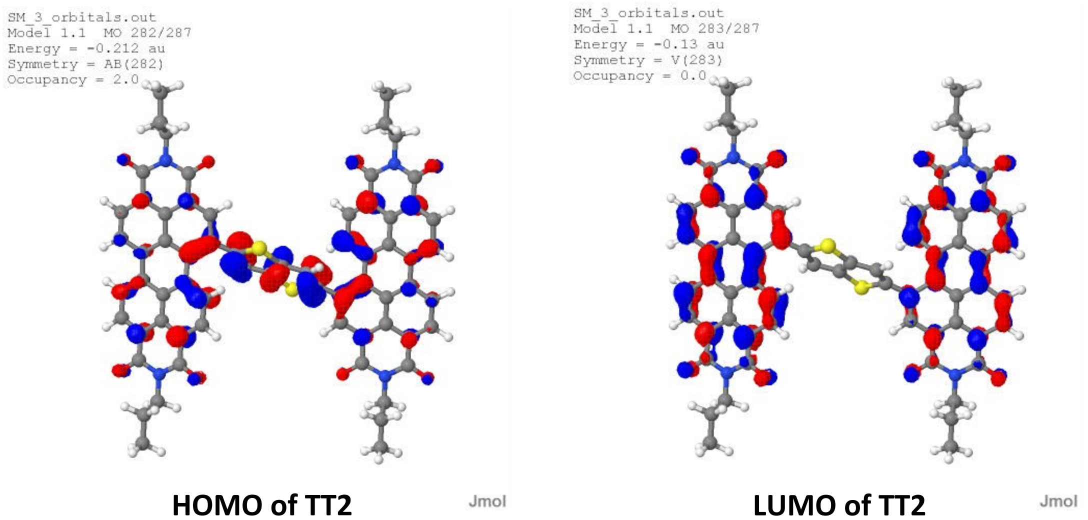

HOMO of TT2 **LUMO of TT2**

Figure S8: HOMO and LUMO orbitals of **TT2** indicating charge transfer character**.**

<u>Low-Lying Anion Excited States</u>

Additionally we compute the anion excited state energies in accord with Troisi *et. al.*[2] and find that twisted ring fusion significantly lowers (~0.2 eV) the energy of the lowest-lying excited anion state, which should help contribute to exciton dissociation in the case of the fused twisted species.

Table S4. Results of DFT calculations on the PDI dimers including Ground State Orbitals, Ground State Absorptions, and Excited Anion State Energies

Species	HOMO-1 (eV)	HOMO (eV)	LUMO (eV)	LUMO+1 (eV)	Excited States (nm) (first 10)	Oscillator Strengths (first 10)	Anion Excited States (eV)
**Ph1**	-6.03	-5.88	-3.51	-3.46	579.4,575.0,.495.9,487.0,411.5,405.6,381.4,381.3,377.0,376.9	.144,.000,.004,1.311,0.472,.000,.000,.0000,.000,.001	**0.73**, 1.33, 1.4
**Ph2a**	-6.13	-6.02	-3.44	-3.36	545.4,530.5,492.8,467.7,435.0,428.2,416.6,415.4,376.0,366.1	.000,.341,.116,.633,.112,.018,.278,.302,.278,.000	**0.5**, 1.3, 1.5
**Ph2b**	-6.24	-5.85	-3.55	-3.31	586.3,555.5,474.3,460.7,443.8,419.2,410.0,379.9,372.3,372.1	.284,.000,.000,.381,.747,.000,.249,.000,.002,.000	**0.73**, 1.51, 1.54
**T1**	-6.07	-5.79	-3.58	-3.47	652.0,611.9,519.0,493.8,443.9,425.6,387.5,386.9,379.9,377.9	.068,.064,.222,.798,.197,.500,.000,.001,.026,.001	**0.68**, 1.24, 1.37
**T2**	-6.14	-6.09	-3.45	-3.34	536.6,519.6,496.7,468.2,450.2,429.9,410.3,399.7,368.9,368.3	.032,.177,.027,.555,.349,.181,.392,.019,.000,.000	**0.57**, 1.33, 1.56
**TT1**	-6.03	-5.74	-3.55	-3.50	649.3,631.8,522.9,521.8,457.7,456.5,451.0,446.2,383.5,383.4	.293,.000,.022,.266,.000,.983,.340,.000,.000,.000	**0.73**, 1.21, 1.36
**TT2**	-6.24	-5.96	-2.47	-3.35	543.7,535.5,499.4,469.1,466.0,451.2,408.0,394.7,389.6,383.1	.433,.000,.260,.000,.000,.675,.296,.00,.00,.591	**0.66**, 1.28, 1.59

Table S5. Specific DFT Calculation Details – Absolute Energies.

Species	Absolute Energy Ground State (Hartree)	Absolute Energy Anion State (Hartree)
**Ph1**	-3359.476822667283	-3362.514843287745
**Ph2a**	-3357.154253676995	-3360.179181384737
**Ph2b**	-3357.173124697952	-3360.200124414167
**T1**	-3680.167228671126	N/A
**T2**	-3677.865941209933	N/A
**TT1**	-4154.302540124171	N/A
**TT2**	-4152.016710246616	N/A

Specific DFT Calculation Details – Optimized Geometries – Ground State

Table S6: Optimized geometry of **Ph1**.
124
Coordinates from ORCA-job SM_2_gr_opt

C	5.60626509707232	-5.70889794647700	-0.17184781458818
C	6.24843072045872	-4.46741331957051	-0.25745912999957
C	5.55626053949045	-3.26222458106092	-0.09227368280787
C	4.14760503898316	-3.29983425763520	0.17842262317257
C	3.49962000338938	-4.57378998549748	0.22127755465228
C	4.23969843748153	-5.77201027620826	0.06085675208404
C	2.09832678501606	-4.64193239621100	0.41015847376331
C	1.36404477581606	-3.47284255322185	0.47301643534391
C	1.96902982665639	-2.19550961030473	0.41901796133138
C	3.38184144434522	-2.09639911201106	0.37786260455089
C	4.13129753204049	-0.83454085063931	0.55840321333263
C	6.23425989628763	-1.96295083298645	-0.19461789665690
C	5.52028010959491	-0.78747946273943	0.19795156170331
C	3.56291762741966	0.31647867566098	1.11891307509623
C	4.27399277661808	1.52040080099830	1.22861648612846
C	5.58121500834558	1.61314196673999	0.77492683749626
C	6.22454480027724	0.45679803617889	0.26660526415748
C	7.56421505769985	-1.83981922538502	-0.61180293192188
C	8.22438999280513	-0.60380830521168	-0.60946860733175
C	7.57653775911182	0.53677358454615	-0.15478392900333
C	6.29925876641416	2.91050913404778	0.85884543888853
C	8.31243953151850	1.82644980282663	-0.10835149232899
C	1.39707393955954	-5.95010997086952	0.48564567591877
C	3.57015141717771	-7.09575552018660	0.13629565395201
N	7.62855314210536	2.93053742820315	0.41634366013980
N	2.18776703730418	-7.09916935289762	0.36638187033217
C	1.51156390402879	-8.40531138204577	0.44720319044172

O	5.76886206080144	3.92161977982730	1.29093449501152
O	9.46358001348356	1.93444429803671	-0.50111705966828
O	0.18960532675271	-6.03396512617384	0.64455740664152
O	4.18371009252025	-8.14351855186466	0.00849830246032
C	8.35061237615242	4.21222007017950	0.48243990884271
H	6.16667637863839	-6.63736748333438	-0.29434438977142
H	0.27716196848032	-3.55058364021517	0.54259119216732
H	7.32059353603276	-4.45686291228822	-0.45239124386876
H	8.11753543581317	-2.71758972675245	-0.94475231229091
H	3.80228408223374	2.40406932871517	1.66255378813298
H	2.54187196829061	0.29462550870204	1.48927457578128
H	9.26092238925684	-0.52572049198865	-0.94189054844642
C	-4.16425229993408	-1.56789206163653	-0.87283808221461
O	-5.65545320273175	-3.97259843439289	-0.91818891354013
C	-6.17501963088506	-2.96742913960249	-0.45993191637502
C	-3.45723589246230	-0.36021787842169	-0.77863699445557
C	-8.19657624394097	-4.28848898535493	-0.00058680367463
C	-5.46309491013944	-1.66558082374586	-0.39641980070428
H	-3.69543346049095	-2.45080797970168	-1.31141022135257
C	-4.02273135439755	0.79017303896319	-0.21401225198136
C	-1.86505039452257	2.16105611516700	-0.10703737479586
C	-6.10426545426107	-0.51020832490272	0.11689433221791
N	-7.48471524501862	-3.00005585076275	0.03693446384936
C	-3.27689161306393	2.05615987408259	-0.04926415244464
C	-1.26708328785201	3.44083855416203	-0.18228657296615
C	-5.40627198959933	0.73842618042215	0.16598499981721
C	-8.17921344652452	-1.88826767917924	0.53106187225490
C	-7.45039323806135	-0.59398531973180	0.55601963518618
C	-4.04586847896172	3.25724136356868	0.15058586884384
C	-6.14701283753387	4.41795113842864	0.60264238157569
C	-2.00630238954409	4.60689400361387	-0.11942028267205
C	-5.45087543403643	3.21485208488821	0.43900418334469
C	-6.12086836724560	1.91298311834421	0.56004514964214
C	-3.40427303067394	4.53366983906875	0.09142506007301
C	-5.51214438809156	5.66177854032185	0.49805282244136
C	-1.31328154329296	5.91793514449885	-0.21896792765692
C	-8.09869675149028	0.54687546760195	1.00951720531713
C	-4.14851156208537	5.72954154340421	0.24957905528939
C	-7.44472814068784	1.78613855830855	0.99517154863825
O	-9.33236149493787	-1.99402025775050	0.91850730757485
H	-0.18178377718969	3.52321145098274	-0.26854348773466
O	-0.11342028639921	6.00653501340325	-0.42582377994879
H	-7.21665967764707	4.40379153808839	0.81056048423066
C	-3.48670881825138	7.05585574632238	0.15443217663062
C	-1.43422056630427	8.37257653757324	-0.16618394973910
N	-2.10209998193777	7.06385289218137	-0.06284635588234
H	-6.07587452111032	6.58856897516349	0.61795720006317
O	-4.10838662881727	8.10152947834128	0.25800702686101

H	-9.13080991966544	0.46614419975455	1.35474198238432
H	-2.44188535316299	-0.33481783111528	-1.16441919143345
C	1.01696190224232	-1.04783007199279	0.31161691509222
C	0.01830877123502	-0.83901542906793	1.28054384137620
C	1.03186583314991	-0.21014809114880	-0.81765701855376
C	0.09072931794389	0.80932845759482	-0.96551776510918
C	-0.90861925705530	1.01752775545428	0.00284285580627
C	-0.92227072526417	0.18080599164070	1.13302802882686
H	1.78616139866639	-0.36217391371005	-1.59374653893486
H	-1.67638134138727	0.33285583747082	1.90923710262726
H	-0.01156860890883	-1.47056846274430	2.17245952185417
H	0.12015739717320	1.44016626759760	-1.85793521995134
C	1.03641032757911	-8.92237208131450	-0.91454705935798
H	0.65715515321112	-8.28292275894825	1.12559394884587
H	2.22628226337502	-9.11021570771605	0.89209910483291
C	0.33673317458062	-10.27775997173305	-0.79877106850317
H	0.00500900869525	-10.63775265951942	-1.78529483927067
H	-0.55299325678949	-10.21716724279583	-0.14951118510551
H	1.00924265869034	-11.04216225088322	-0.37365242046080
H	1.90597376582324	-9.00441388123956	-1.58857480980473
H	0.34947389855967	-8.18193498451353	-1.35858849769226
C	8.20498849557942	5.05238999529385	-0.79097064782801
H	9.40780627986595	3.97774729164480	0.66269173115889
H	7.95203807035623	4.76086071804612	1.34582215506357
C	8.96273031555299	6.37709801541148	-0.69301253481489
H	10.04209209970809	6.21219961948831	-0.53478709923135
H	8.59236882903543	6.99118021168939	0.14568166083964
H	8.84678367198164	6.96836235884546	-1.61561627618517
H	7.13386841118149	5.24411045076809	-0.97221714016584
H	8.57928935611399	4.46864303166144	-1.64914093481232
C	-1.38370045721711	8.90954909693830	-1.60042210798961
H	-0.41734630240760	8.24788265516146	0.22828448359110
H	-1.98692870328245	9.06647877941449	0.48041458790037
C	-0.69219713732472	10.27168346411802	-1.67775376819724
H	0.34750104731261	10.21513488938882	-1.31410522700935
H	-1.21825658156062	11.02490264001325	-1.06724662584408
H	-0.66306134969495	10.64351530636982	-2.71436432431523
H	-2.41271411709387	8.98798812303252	-1.99000848261979
H	-0.85147389833328	8.18205653679951	-2.23615798218948
C	-8.96692426098331	-4.51491055885472	-1.30579119259051
H	-8.88683278298168	-4.29904006153875	0.85342912196180
H	-7.44295149890883	-5.07498954587393	0.13628325141232
C	-9.69774976861691	-5.85813002701126	-1.31307349758187
H	-10.42669495622246	-5.92472859950741	-0.48738649778024
H	-8.99306626701318	-6.70000749674965	-1.20477784870002
H	-10.24831529591220	-6.00484958018448	-2.25593332800315
H	-8.25944998602490	-4.46781497803963	-2.15123477621336
H	-9.69080966390398	-3.69368247170985	-1.44172324615420

H -7.99823621324759 2.66349196171072 1.32889199027778

Table S7: Optimized geometry of **Ph2a**.

120

Coordinates from ORCA-job SM_5_gr_opt

C	7.76077328534480	-1.52296632354361	0.18617330643463
C	7.17395483788712	-0.30842045750311	0.54613165911063
C	5.77957590279945	-0.16490313191208	0.64974783831036
C	4.95473736275777	-1.29508694921683	0.35700425548736
C	5.56563800646041	-2.53752769751612	0.02087574639094
C	6.96866041977690	-2.64317551512667	-0.06702482956367
C	4.74736426969207	-3.68006133433693	-0.22201953306517
C	3.37707071593279	-3.58851428023701	-0.13573311753837
C	2.72685525684976	-2.35692990031124	0.16597894206901
C	3.52465349342009	-1.21018191584130	0.40690606342253
C	2.89715375503045	0.02841633152534	0.76325904265076
C	5.14124157264538	1.08296813638462	1.08001284312609
C	3.71465238838493	1.14237277663520	1.15215019704748
C	1.48052467466377	0.13996642547468	0.75734662701795
C	0.90314607393893	1.31346611794149	1.33243495379215
C	1.67090919628984	2.37590684504193	1.74478142981105
C	3.09119393568222	2.33233188120918	1.62456027306663
C	5.87919148567225	2.22271343771570	1.44543557153087
C	5.25594962783351	3.38593360734786	1.89945526152333
C	3.86578109343521	3.44612432973031	2.00404973807901
C	0.99970270801107	3.57393054202524	2.31728538925749
C	3.21802803962903	4.67509059475557	2.52929947649159
C	5.36541336080173	-4.98841860970542	-0.57033413721637
C	7.61057888215367	-3.93405443129447	-0.42205729809750
N	1.82296860500174	4.64700290515224	2.67928167385864
N	6.76295237928186	-5.03273356045586	-0.63093820969826
C	7.39010302045283	-6.32047812010986	-0.97527540044001
O	-0.21107472568568	3.63763030733967	2.45866505036080
O	3.85739643558850	5.66860573800430	2.83500753813819
O	4.69672151515848	-5.98537257059367	-0.79327004315628
O	8.82070658988516	-4.05216920639775	-0.52788693622732
C	1.17220669989368	5.84770845308576	3.23144293814968
H	8.84568099386129	-1.61698615262258	0.10859043553232
H	2.80587490169673	-4.49400430602607	-0.33678105038392
H	7.82791537780317	0.53796635944998	0.75604016461091
H	6.96779863760577	2.20979764324472	1.38864078402386
H	-0.17243355375988	1.38913420918257	1.47975000042585
H	5.84469846105196	4.25812298020874	2.18954127854596
C	-3.83590721914060	-3.02897950142692	0.52617471653802
O	-5.50296316756717	-5.18066811648973	1.24498716372899
C	-6.00966460246412	-4.09189000669728	1.02422676412611
C	-3.00823224333925	-1.91976359063413	0.18691891303530
C	-8.20371673737113	-5.07128564822565	1.53632460229790
C	-5.20252134273938	-2.90540389598146	0.62995407347350
H	-3.40739178114122	-4.00608409165867	0.74436861712807
C	-3.62356011427855	-0.67080224819494	-0.07914982770509

C	-1.40130779950311	0.34075226531736	-0.47980339234961
C	-5.83846273846862	-1.65664921345998	0.36513256357941
N	-7.39241013695353	-3.90846949859812	1.13708982266346
C	-2.81807480081622	0.44726790583484	-0.47396286170505
C	-0.65686264108283	1.39526552742859	-1.09167654368530
C	-5.04879261477910	-0.53315434091609	-0.01452186087648
C	-8.06787197939934	-2.70846822478459	0.86713852228890
C	-7.23977754220587	-1.54253510923064	0.46828810179270
C	-3.45928938299516	1.66322446184914	-0.88701016795279
C	-5.43554883802413	3.05562222383390	-1.19254495798288
C	-1.25688220694356	2.55311288135040	-1.52511763762868
C	-4.87708578858715	1.82559014430835	-0.80193612704857
C	-5.69372722087314	0.70262459096736	-0.33108348563621
C	-2.66550869187618	2.73191184009320	-1.39250863494391
C	-4.64645117245343	4.09675044904552	-1.68307035566359
C	-0.41574562625150	3.62134383848520	-2.12892100677850
C	-7.85349395121071	-0.32100243030659	0.18995599149850
C	-3.26451412183199	3.94114316611110	-1.79687387612744
C	-7.09192306363407	0.77874679054893	-0.20919185273428
O	-9.28277413631773	-2.64283388942615	0.96211005375091
H	0.41581168506739	1.29892047740717	-1.24905276147276
O	0.78444472791959	3.48633805266507	-2.30502406928580
H	-6.51227097464505	3.21324662943949	-1.12657531550602
C	-2.44171977947242	5.04419920780807	-2.35455043451700
C	-0.23885463096065	5.89526637437496	-3.04247588757192
N	-1.06207382028237	4.81521355365670	-2.47237596208585
H	-5.09772335641305	5.04098362234555	-1.99295825346367
O	-2.92868560797476	6.10638409334625	-2.70644557192421
H	-8.93878111003847	-0.24400282657452	0.28054409716094
C	1.28073333247690	-2.24710487773526	0.18864472726522
C	0.66522561597805	-0.97003500826328	0.29009489386632
C	-1.56236414459842	-2.03188244301556	0.15587443713134
C	-0.91408852452547	-3.30078059437307	0.23109665645483
C	0.44815829334372	-3.40529939955293	0.14679326438424
C	-0.76075718224812	-0.86607132481759	0.01749052057182
H	0.89719267828815	-4.39720705266658	0.11633236926129
H	-1.50644360796157	-4.21309345480252	0.29103467188592
H	-7.61109856532548	1.71026394258358	-0.43449452235193
C	7.57191437173861	-6.51741699252558	-2.48347041554818
H	6.74860421953965	-7.11065861037459	-0.56349309129964
H	8.36295302277577	-6.34816873379574	-0.46800760657635
C	8.21762197690365	-7.86477328574198	-2.81021669629740
H	8.34215175051462	-7.99200813457779	-3.89723468209482
H	7.60315807404902	-8.70449770498682	-2.44369843949338
H	9.21455874640452	-7.95566318121343	-2.34760076175699
H	8.19399204128933	-5.69675368251820	-2.87891517115746
H	6.58743825851151	-6.44460878079064	-2.97548451161009
C	-8.65791014113285	-5.92651103638898	0.34911824061658
H	-9.07530758055420	-4.68180301101101	2.07771391626349
H	-7.59179453576516	-5.67121311355498	2.22269436127776
C	-9.50492984760609	-7.11942056311401	0.79441238295237

H	-8.94275895434813	-7.77876746285478	1.47763585953752
H	-9.82020413545958	-7.72529871810472	-0.06967543108943
H	-10.41605045089763	-6.79099614185138	1.32212828951008
H	-7.76779278151209	-6.28056852713044	-0.19797792024876
H	-9.23546798511391	-5.29404010687271	-0.34649414576193
C	0.75081247322892	6.85397090198971	2.15589181022209
H	1.88702639606521	6.31059193921380	3.92435391538311
H	0.29443478080439	5.50662258921035	3.79589747996028
C	-0.11465265268166	5.81928004838969	-4.56759066010240
H	0.75268564779785	5.82324997935326	-2.57707149082953
H	-0.70489613301974	6.84418010571402	-2.74673563706618
C	0.74454606419193	6.95298529646838	-5.12816976683351
H	1.76658862736813	6.92495704476274	-4.71477312703096
H	0.31565610030240	7.94027339807608	-4.88569474040752
H	0.82594803018704	6.88408609096974	-6.22459079346843
H	-1.12364067815492	5.85723203548557	-5.01184571841220
H	0.32316509429087	4.84470304373064	-4.84280614559362
C	0.08071728174008	8.08919726180545	2.75959102891617
H	0.76011032335056	8.61652002426433	3.45091105889914
H	-0.82650115496056	7.81845781694092	3.32570478797942
H	-0.21603273547484	8.80189520108017	1.97407649287887
H	0.06091914175262	6.35717281264805	1.45236003538680
H	1.64165829456676	7.15388178763188	1.57847919312972

Table S8: Optimized geometry of **Ph2b**.
120
Coordinates from ORCA-job SM_6_gr_opt

C	7.37846903225957	-2.92420419995518	0.20351158919703
C	7.40084427611431	-1.52763292143875	0.20518206646423
C	6.21945421745438	-0.76893692575879	0.20285306875918
C	4.96434798904489	-1.45500217995307	0.19938737863917
C	4.95559714846603	-2.88084240755369	0.19930301769784
C	6.16537243843058	-3.60819563802743	0.20072792785293
C	3.71309936608903	-3.57456331627494	0.19344497490168
C	2.52729965870682	-2.87605030209515	0.19043339453060
C	2.49255612190665	-1.45461043992203	0.19302919418516
C	3.71760994893040	-0.74364660246625	0.19661989835036
C	3.72573558576485	0.70088410529066	0.19667955941996
C	6.22769211112055	0.69794365876700	0.20313715139194
C	4.98037014442657	1.39813428499130	0.19966717484483
C	2.50876470894707	1.42563966724880	0.19302969430720
C	2.55948592922716	2.84658707336442	0.19036734991135
C	3.75305294713656	3.53170789522541	0.19370058015709
C	4.98765182771519	2.82398103762434	0.19984891495054
C	7.41755319862510	1.44330959144371	0.20591714783580
C	7.41086324684615	2.84004509429104	0.20463831769601
C	6.20552910701943	3.53762822009152	0.20174442705211
C	3.73310459930232	5.01960230101367	0.19083822436681
C	6.21608621885875	5.02260509446240	0.20058182829985
C	3.67651557516267	-5.06213979500299	0.19048699099645

C	6.15932888354727	-5.09321647285799	0.19929421327818
N	4.97228194576974	5.66962739438378	0.21364692546666
N	4.90827991461496	-5.72613453408848	0.21299446557836
C	4.88934116496577	-7.19859933519554	0.21274514776475
O	2.69212731865371	5.65664888100244	0.17191514946523
O	7.25264266304733	5.66710961122668	0.19074865703668
O	2.62843724322181	-5.68749655539847	0.17196316123563
O	7.18858305672069	-5.74925878524354	0.18867799227946
C	4.97043168250650	7.14219497800862	0.21335313218493
H	8.30771601074171	-3.49689153020277	0.20445896262154
H	1.61100305901859	-3.46447874934510	0.18536970833607
H	8.37074825981240	-1.03105163663423	0.20816185688063
H	8.38192774021539	0.93596972143832	0.20860975902903
H	1.64991615403177	3.44536768560932	0.18484774211942
H	8.34645110821716	3.40231312367011	0.20592993249923
C	-2.56047012513410	-2.84705498872890	0.19075107950167
O	-2.69312146684143	-5.65712245057005	0.17268277964385
C	-3.73410302165966	-5.02007575726288	0.19150878384673
C	-2.50974086101627	-1.42610508416907	0.19330243406583
C	-4.97145104469679	-7.14265601394868	0.21398950429785
C	-3.75403798700179	-3.53216917843029	0.19417800000991
H	-1.65090219729913	-3.44583667019217	0.18526190586728
C	-3.72670699223403	-0.70134264061907	0.19699581906020
C	-2.49351802091511	1.45414538507653	0.19321038461239
C	-4.98863360760389	-2.82443194866126	0.20030313017933
N	-4.97327928337755	-5.67008861929557	0.21422469739906
C	-3.71857376152860	0.74318833038234	0.19689650474517
C	-2.52825065152740	2.87558472297908	0.19060302616444
C	-4.98134594656951	-1.39858500488029	0.20007103636394
C	-6.21707482755722	-5.02306167948407	0.20106333814672
C	-6.20651465909932	-3.53807295780360	0.20223170629352
C	-4.96530619039662	1.45455193128559	0.19973614624843
C	-7.40180434494374	1.52720326763682	0.20568893388546
C	-3.71404299737294	3.57410845219556	0.19369498887569
C	-6.22041967931481	0.76849724087169	0.20328375324407
C	-6.22866474484524	-0.69838635990461	0.20357487604733
C	-4.95654472539236	2.88039259028227	0.19964690906791
C	-7.37942505255124	2.92377756692928	0.20402183148830
C	-3.67744551779922	5.06168779228330	0.19071294345483
C	-7.41184332317755	-2.84048550906567	0.20512685753833
C	-6.16631788904426	3.60774978168156	0.20115987988540
C	-7.41852833549133	-1.44374763478991	0.20639498074378
O	-7.25363285252520	-5.66755766128257	0.19125997351143
H	-1.61194717063849	3.46400141555510	0.18545943529376
O	-2.62935857879282	5.68703527784894	0.17215437851822
H	-8.37170910094496	1.03062298501046	0.20873763707896
C	-6.16026363300247	5.09276956840060	0.19976855098787
C	-4.89026946974102	7.19814681519690	0.21321309260615
N	-4.90919460453172	5.72568352647027	0.21338831190163
H	-8.30864991200880	3.49650422699467	0.20504383018483
O	-7.18950710825981	5.74881337073784	0.18918314525246

H	-8.34743585928637	-3.40274750848080	0.20642533394253
C	1.22898176279541	-0.72374665742630	0.19192681554539
C	1.23703419492589	0.70910008899674	0.19193069680322
C	-1.22994979207761	0.72327647257417	0.19201977081172
C	-1.23800560307352	-0.70957045076988	0.19206608793735
C	-0.00832359121751	-1.38286705439897	0.19170578586953
C	0.00735455886778	1.38239675331726	0.19167211516196
H	-0.01491698430829	-2.46868333394491	0.19214777247195
H	0.01394724732822	2.46821401758243	0.19209754505613
C	4.89099431024463	-7.79939441454259	-1.19645857875419
H	3.98687759660243	-7.50786075622047	0.75598459455486
H	5.77522665055819	-7.53167178488362	0.76906730341537
C	4.86944803967129	-9.32782616255334	-1.16765784705165
H	4.86946896233157	-9.74423679898758	-2.18707221493727
H	3.97099724150739	-9.70826903874108	-0.65246656151640
H	5.75055345900704	-9.73341108685078	-0.64182145569093
H	5.78693092700601	-7.44603874886742	-1.73513181088399
H	4.01259770623507	-7.42154883451290	-1.74720293237569
C	-4.98753802603260	-7.74340512365020	-1.19514301483185
H	-5.85817106631240	-7.46548488719022	0.77507065701499
H	-4.06971501848643	-7.46223964457112	0.75236630636619
C	-4.98593705271531	-9.27199486886095	-1.16629310245818
H	-5.87033729441396	-9.66580638107762	-0.63703396948815
H	-4.09064710456720	-9.66435775816221	-0.65454991423001
H	-4.99546256862250	-9.68841447530975	-2.18565951935549
H	-4.10682788462293	-7.37719019520453	-1.75016256396282
H	-5.88126462378973	-7.37830927699313	-1.72950461899584
C	-4.89200155041283	7.79902186139686	-1.19595666422193
H	-3.98779829807177	7.50741159132537	0.75644239220266
H	-5.77613813289166	7.53116978869193	0.76958915029710
C	-4.87057791335332	9.32745347382956	-1.16707018286680
H	-3.97216936669763	9.70793185630105	-0.65183273214234
H	-5.75173111580689	9.73294221880205	-0.64123869729921
H	-4.87060801290585	9.74392212753673	-2.18646194009100
H	-5.78792399068908	7.44562522410812	-1.73462688689211
H	-4.01358959052500	7.42127553537330	-1.74674587839083
C	4.98645807844646	7.74289487853089	-1.19580119247348
H	5.85717072310347	7.46504945223160	0.77438731202174
H	4.06871422202305	7.46179080556420	0.75175637764050
C	4.98485529989395	9.27148515933646	-1.16699206242950
H	4.08959094165786	9.66385439087889	-0.65520875160898
H	4.99433317608284	9.68788279838697	-2.18636742098276
H	5.86928112425458	9.66531281863721	-0.63778753981918
H	4.10572777865484	7.37665943063855	-1.75077215767578
H	5.88016520242886	7.37778723897677	-1.73018893615906
H	-8.38290068054984	-0.93640392128605	0.20910186687560

Table S9: Optimized geometry of **T1**.
121
Coordinates from ORCA-job SM_1_gr_opt

C	7.63665112378821	-2.31208157994095	0.26037088836558
C	7.35626162827165	-0.98547827231764	-0.08990271402561
C	6.04703539646729	-0.49344425838279	-0.13759693785501
C	4.96016445655861	-1.37125654337734	0.19051176161845
C	5.26079883142384	-2.73495573394886	0.49567511833069
C	6.60092759520400	-3.19083831485101	0.54687580740348
C	4.20460828747658	-3.64813613940430	0.73295885662038
C	2.89856310183622	-3.23227882240143	0.57266536280861
C	2.56226570222891	-1.89111724295783	0.25679624189576
C	3.59410883724626	-0.91618572020876	0.19193296268862
C	3.37238091484958	0.54597780060175	0.14834694470449
C	5.75711194201815	0.89439064351243	-0.52095916265179
C	4.43810639173287	1.39872547531378	-0.29576662190136
C	2.18667726165456	1.15696466209539	0.57695119636619
C	1.96978597170452	2.53626374263024	0.44424721242478
C	2.93641756147965	3.35129026728796	-0.12975449814621
C	4.19166830989245	2.79620310016266	-0.48245944947606
C	6.73179011662072	1.75373220736509	-1.03982080404363
C	6.45882627130329	3.10526895474976	-1.29416072937485
C	5.21078498481223	3.63719313860403	-0.99954390619895
C	2.65458092775766	4.79447890773876	-0.34184166644515
C	4.96101526290089	5.08507494819456	-1.21790332318058
C	4.48144437489676	-5.06571681556946	1.08450389101495
C	6.91154945438039	-4.59922635003974	0.90206707019809
N	3.70012514165046	5.57542162448956	-0.85502352200958
N	5.82591701498450	-5.44346281235977	1.17290859814873
C	6.12184021099681	-6.83853611084614	1.54067960712014
O	1.56972354994555	5.29170911345604	-0.08703041866748
O	5.80731456464199	5.82882403133424	-1.68935453074578
O	3.58835933486697	-5.87199768618077	1.29058007482987
O	8.05656908264914	-5.01742947832020	0.96472855994809
C	3.44337398043289	7.01081009061254	-1.05988924048989
H	8.66591842810290	-2.67293984553559	0.30378346335706
C	1.14910346714444	-1.63546321284739	-0.08650638337673
H	2.10921628268805	-3.98023578115277	0.66825102428509
H	8.19329113956781	-0.32633572038114	-0.31988591430770
H	7.73391260432356	1.38172359644515	-1.25184694624562
H	1.03303183462952	2.99039053661535	0.77373261956685
H	1.39719308471679	0.55152613083617	1.01604464383259
H	7.22972013318693	3.76207460703200	-1.70101422034818
C	0.63699650871443	-0.93761815553901	-1.16187869609659
C	-0.78102815092717	-0.94640266817350	-1.22347549044191
C	-1.37354794641339	-1.66579864884492	-0.20546288507888
H	1.26835781834074	-0.43419809163551	-1.89555365037054
H	-1.35843671740918	-0.45000311659692	-2.00522903326108
S	-0.15700088014242	-2.35406055963326	0.84067347521518
C	-3.30678746867096	-3.21250704026475	-0.20218411897603
O	-4.32767805283971	-5.83216198476795	-0.47401764952068

C	-5.10887519596979	-4.89427281294640	-0.47147000759283
C	-2.82709134740714	-1.89310167772214	-0.02703865979082
C	-6.94710369777423	-6.49212445992912	-0.79897978690197
C	-4.65910762542189	-3.48848530899559	-0.29706491070597
H	-2.59794580194189	-4.03711122317874	-0.29964367088717
C	-3.74891240781464	-0.83433688280283	0.15930217320529
C	-2.18909562298194	0.77258409464391	1.32236670934764
C	-5.59296104940218	-2.42188603912200	-0.29920805251813
N	-6.48393435478836	-5.10351866030339	-0.63499541357917
C	-3.37388711549367	0.51308063954225	0.62390099778589
C	-1.82913505927950	2.07033342983180	1.70908667175088
C	-5.13952341944658	-1.08184514209784	-0.09973106102047
C	-7.44620722232413	-4.08609622472909	-0.69653644559835
C	-6.96594231035526	-2.69143407783125	-0.52179724420564
C	-4.28673162662330	1.59770321136493	0.41781071865821
C	-6.42268265012413	2.48727573737430	-0.33723500049644
C	-2.62939982837109	3.15321521151346	1.37520516278477
C	-5.61473030295584	1.37784203140253	-0.06573415747752
C	-6.08677872922225	-0.00839750969172	-0.20075461374545
C	-3.87483295656951	2.92885881780444	0.73825136775827
H	-1.52524163770786	-0.04540041068172	1.59110144767658
C	-5.97373683200285	3.79782406929749	-0.11069465139544
C	-2.19113173469696	4.52686625453621	1.72722603198578
C	-7.87056413568913	-1.63973268464756	-0.58314075164062
C	-4.72099056264674	4.02764435314146	0.44242865405329
C	-7.43075114947677	-0.31687237899312	-0.43683785247358
O	-8.62338079172955	-4.35065636129633	-0.88226949268629
H	-0.90660058469029	2.24774586297256	2.26488180891248
O	-1.13967615324971	4.74329475391030	2.30758738294969
H	-7.42838463260161	2.34986702825910	-0.73518446290270
C	-4.27547447656063	5.42012044633260	0.71494511598186
C	-2.56591807378564	6.94908142174815	1.61707704483780
N	-3.03551410979269	5.57867354790145	1.34933154891242
H	-6.61259453683519	4.65146422422585	-0.34423030776916
O	-4.95097096502789	6.38908780322433	0.40656466208822
H	-8.92562593687634	-1.85948816493095	-0.75754434305809
C	6.22445154443531	-7.77377059911030	0.33175450641042
H	5.31688145844278	-7.17073233299032	2.20937915362876
H	7.06949590855088	-6.82974167230602	2.09498481949561
C	6.53306088166326	-9.21294062765542	0.74730516060308
H	5.74703263364557	-9.61890331289381	1.40659862746407
H	7.49030975515397	-9.27708972356395	1.29217906601327
H	6.60510057380000	-9.87177084671679	-0.13233414729315
H	7.01304169140452	-7.40107686707259	-0.34435020446184
H	5.27416335330036	-7.73938121621700	-0.22730149360902
C	-6.96304104321908	-6.95402659928075	-2.25954046663187
H	-7.95740770857328	-6.54499468764728	-0.37245868013999
H	-6.27443298085175	-7.12636493842579	-0.20714562908889

C	-7.44548851272104	-8.39874894738052	-2.39661003094082
H	-6.79406178178801	-9.09483509583570	-1.84203646833134
H	-7.45192942734442	-8.71644601334219	-3.45131295392126
H	-8.47025732037869	-8.51942157042073	-2.00639807843391
H	-5.94649270472489	-6.85778323223951	-2.67728875355275
H	-7.61760255825242	-6.28242917567700	-2.84004616806600
C	-1.66233387230034	7.49555773349540	0.50583211566735
H	-2.02139731975029	6.92409926631953	2.57049079674647
H	-3.45874052024990	7.57689787945944	1.73024800192566
C	-1.17772776602591	8.91356494485330	0.81222309579168
H	-0.59285268513714	8.94359283935293	1.74730801557458
H	-2.02206037986667	9.61484039993545	0.92447954268134
H	-0.53253042445433	9.29490138874951	0.00424741935725
H	-2.22358333692418	7.48871813615770	-0.44455813996393
H	-0.79763809271540	6.82328753923702	0.37914348003232
C	2.90060141317998	7.33573348138528	-2.45519027754258
H	4.39396403054087	7.53413983768776	-0.89257736475784
H	2.72297456179588	7.31934829025514	-0.29131601475689
C	2.64601552275923	8.83277056813414	-2.63475000675750
H	1.90888676228690	9.20685354608003	-1.90359605539086
H	2.25633704874631	9.05154729032092	-3.64166581737873
H	3.57209770371759	9.41734422472417	-2.50261463677900
H	1.96518354504890	6.77311363352677	-2.61497273491676
H	3.62357974357061	6.98476652670604	-3.21075421955778
H	-8.17025473081383	0.48148014067008	-0.50358493887021

Table S10: Optimized geometry of **T2**.

117

Coordinates from ORCA-job SM_4_gr_opt

C	7.46954325132904	-2.48682878575525	-0.34523199831442
C	7.17816291841315	-1.14596511716574	-0.09434628959160
C	5.85742044841941	-0.70097246523817	0.09420492967256
C	4.79945066824329	-1.65745050074108	0.00435295841868
C	5.10943370730240	-3.02989607892223	-0.22904615330298
C	6.44695927244372	-3.43563250935939	-0.40424457582822
C	4.05893526545825	-3.99826227664426	-0.28184369411885
C	2.74921499906458	-3.61396458240951	-0.12263964325856
C	2.40388199427400	-2.24650889679666	0.07992427960707
C	3.42971929930401	-1.26554122585006	0.15183803504543
C	3.09582346340143	0.10078734781112	0.42063007473620
C	5.52497275781518	0.69152512917347	0.40912156232578
C	4.15411436482029	1.05356793197000	0.59773500313024
C	1.73287015532984	0.50742765807827	0.54278497137905
C	1.47018329179573	1.81912074682926	1.03722309098642
C	2.47406100383478	2.73710394308722	1.23027312250697
C	3.83288744378568	2.39012193335182	0.97169515174300
C	6.50648473026387	1.68867607606467	0.55264674476937
C	6.17699942497245	2.99918932242278	0.89955147698152
C	4.84656688632780	3.35601267733259	1.12263744887236

C	2.11994616989182	4.10233618489883	1.70514984065418
C	4.51312787122279	4.74696377662578	1.52313665344003
C	4.37006704658783	-5.43603331747639	-0.51251324705244
C	6.78319097942438	-4.86196034907360	-0.64530808289427
N	3.16821113723799	5.02475636421820	1.80829551108314
N	5.71907527536736	-5.77620481364475	-0.66009642688765
C	6.04423031979751	-7.19629891141304	-0.88013858754864
O	0.97421746452368	4.41981822392891	1.97885212326815
O	5.36244086322383	5.61859949188242	1.61410059914186
O	3.49844046121457	-6.28839164237599	-0.56870649374583
O	7.93046010348844	-5.23977311661682	-0.81842213026208
C	2.82697763185279	6.39949224068914	2.21089669348687
H	8.50040468288021	-2.81574936769350	-0.48911508465394
H	1.97561018761606	-4.38310325307788	-0.17655807593388
H	8.00743936388658	-0.44054356316944	-0.03941333732834
H	7.55808810036758	1.44590846949773	0.39920416681987
H	0.45910316927852	2.12257635467077	1.29955664826419
H	6.95264556290242	3.75875099184231	1.01270344749086
C	-3.47640117332280	-2.91792401691459	0.50698835141572
O	-4.80179672883554	-5.36529656572370	0.90958247616390
C	-5.46222985047573	-4.33963197657122	0.87166330016127
C	-2.83610188687977	-1.65910229997470	0.31727319132874
C	-7.47988932925573	-5.68548992065043	1.25841357506476
C	-4.84011233902183	-3.00482761796339	0.65198834033721
H	-2.89229253832482	-3.83955560770477	0.56084661013787
C	-3.61979893109875	-0.47574592522664	0.24209554083243
C	-1.57005297209785	0.88056254285412	-0.12722545840647
C	-5.65098794842142	-1.82868292010801	0.59551753934122
N	-6.85083827086389	-4.37157189232053	1.03735782026262
C	-2.99035115891750	0.78433897489886	-0.01705254039722
C	-1.02112766697483	2.10422903203815	-0.61505487688974
C	-5.04391637542250	-0.55751876993167	0.37248401514954
C	-7.69225692723987	-3.25025831703683	0.97609898538980
C	-7.04752300520702	-1.93154081913443	0.74864024051996
C	-3.81198799657047	1.94654503240966	-0.20349500047288
C	-5.96992525441417	3.08044977913598	-0.18888949556504
C	-1.79689922097255	3.22066070317601	-0.81617208564843
C	-5.23116179438064	1.89376343489407	-0.03213506613313
C	-5.86480315463520	0.60807811373983	0.27463961581625
C	-3.20177542275919	3.17996095804153	-0.57298272862210
C	-5.35875474176110	4.28500732018944	-0.53801602289471
C	-1.14830821789706	4.47401811435564	-1.29068910337742
C	-7.83599014020752	-0.78102551421965	0.68156246136087
C	-3.97974021969114	4.34154548860327	-0.74533501739566
C	-7.25347952997561	0.46407044496883	0.44420365157267
O	-8.89966187221096	-3.36990928694253	1.10576906947177
H	0.03350028156150	2.17839865834187	-0.87005981004605
O	0.04061913697672	4.53180644118311	-1.55928123193258
H	-7.05075748169955	3.07301819166340	-0.04728162341397
C	-3.34971340640323	5.62027428323613	-1.16303646014310
C	-1.33940633374350	6.86151786599709	-1.84149265432357

N	-1.96743448743580	5.60412000316803	-1.40211301662007
H	-5.94882044552531	5.19422663691727	-0.66712370992616
O	-3.99469408109812	6.64620704739279	-1.30711653648149
H	-8.91600841115711	-0.87635880412727	0.80916341363150
C	1.05323906894556	-1.80439036249426	0.17442378793887
C	0.68712253587449	-0.44955785540034	0.26387468449154
C	-0.76315320258556	-0.28575901245943	0.15147530252388
C	-1.42075663788400	-1.52625387655011	0.23327753204605
S	-0.32081675639064	-2.88490859158222	0.20225470153272
H	-7.90527025271527	1.33545947492913	0.38277176214498
C	6.04910334977756	-7.59343776916467	-2.35941162715600
H	5.29800834857253	-7.78491923537078	-0.33085950297555
H	7.03396555565747	-7.36989212757829	-0.43760535764685
C	6.39693595574316	-9.07026165023216	-2.55181950653434
H	6.40157009815315	-9.34096938885586	-3.61940221418537
H	5.66904125248108	-9.72488224615967	-2.04375527079690
H	7.39514792611986	-9.30415388527344	-2.14432539909067
H	6.77664478653518	-6.96242455715419	-2.89712134841813
H	5.05587210070262	-7.38239195632941	-2.79087035919651
C	-7.86040547409806	-6.39656366884190	-0.04431576433440
H	-8.37319445051171	-5.51444454538668	1.87304039927481
H	-6.76380064864406	-6.29362970416198	1.82664965523308
C	-8.51710264930645	-7.75356113310610	0.21544323795492
H	-7.84169235209016	-8.42777134581529	0.76919528042290
H	-8.78393690847095	-8.25108295057908	-0.73026414277032
H	-9.44126609262808	-7.64606435441148	0.80814495293079
H	-6.95322981096192	-6.52871526391313	-0.65813840238672
H	-8.54717850130823	-5.75010169762396	-0.61684998800620
C	-1.34568653158176	7.03950396503232	-3.36292102887908
H	-0.30802465753582	6.85268962212050	-1.46525808783097
H	-1.88938116563858	7.68052019105770	-1.35994582162116
C	-0.68649887644492	8.35267856961890	-3.78724971962332
H	-0.69675219857489	8.46625820906410	-4.88301854849047
H	0.36484708334868	8.40063175038873	-3.45664531995768
H	-1.21017243604808	9.22263196933130	-3.35637185137675
H	-2.38813715601897	7.01061409286319	-3.72249547473756
H	-0.81859427912007	6.18793777968625	-3.82497601760521
C	2.48741741121460	7.29988529152122	1.01841151832146
H	3.69162682084080	6.80106488757852	2.75506179479360
H	1.96998654306388	6.33219610649685	2.89391200815292
C	2.11681612608481	8.71724336839197	1.45736163757991
H	2.94331583223093	9.19574750698608	2.01010288035195
H	1.23170880145538	8.71592669602374	2.11630649395826
H	1.88561495552679	9.35337179554874	0.58838328014905
H	1.65328204064410	6.84587941585296	0.45659911728752
H	3.35510260458216	7.33172358234593	0.33755425416052

Table S11: Optimized geometry of **TT1**.
124
Coordinates from ORCA-job SM_3_gr_opt

C	8.12218287394854	-3.58993559743009	-0.30246077750444
C	8.23797311762043	-2.19755954577655	-0.39756578118351
C	7.14359636883962	-1.34862230149684	-0.19683438104224
C	5.86777799964122	-1.92202807499274	0.12269240082111
C	5.75717560956404	-3.34684273219280	0.17545715076703
C	6.89292692381207	-4.17076378963355	-0.02285613610869
C	4.49580411725078	-3.94557239706242	0.41399914253178
C	3.37185160202876	-3.14830849673842	0.50718021713291
C	3.44373465403842	-1.73546676976079	0.44068259115275
C	4.70996836141666	-1.10197065187398	0.36576774139103
C	4.92875314972060	0.34814344427391	0.55076205018983
C	7.26705180136245	0.11115329700816	-0.30749491082177
C	6.17698208583584	0.92335074348309	0.13718633466271
C	3.99430867985916	1.18912891050741	1.16875008312405
C	4.19855752019453	2.57156970102385	1.28126426934702
C	5.34606853837849	3.16079545993910	0.76984716697082
C	6.35546912766464	2.34188258010431	0.20475138445020
C	8.42767767857204	0.73459829633624	-0.77853948993678
C	8.56416793854454	2.12967441472698	-0.77969263597037
C	7.55256513622101	2.93442312715308	-0.27337212630331
C	5.51673685322032	4.63371262725380	0.85072174290266
C	7.74046090741379	4.40732864338604	-0.23068000559402
C	4.35314021483224	-5.42252046818755	0.50490059240519
C	6.78469096605566	-5.64978357221327	0.06514509546235
N	6.71345010548408	5.16309549394460	0.34919550046606
N	5.51905570153024	-6.18096394202273	0.34798079075876
C	5.40135712370717	-7.64561573121580	0.45107843704729
O	4.66297764152815	5.36367333669182	1.32897258423065
O	8.74170946325392	4.94960477457842	-0.67184127820541
O	3.27724493066114	-5.96194778860807	0.70811632955769
O	7.74663017939056	-6.38392621262000	-0.09553211891947
C	6.89191299223956	6.62365092241249	0.41042147189764
H	8.99077201179360	-4.23354666714105	-0.45301377943459
H	2.39968879406376	-3.63414653392899	0.60905942033158
H	9.21735196880871	-1.77784788534759	-0.62617792559630
H	9.25877865761905	0.13638419116243	-1.15155519403931
H	3.44740600177565	3.20306722112297	1.75922474305376
H	3.07795488101061	0.77419439703559	1.58039603926273
H	9.47572887888348	2.59927351908887	-1.15405303952673
C	-4.03820840865433	-2.57631476324913	-1.00484214357064
O	-4.51765402262057	-5.36616195251110	-1.07595296465385
C	-5.36467267276361	-4.63574437981075	-0.58640605646092
C	-3.83103014509772	-1.19464840826230	-0.88768024605318
C	-6.73095538333275	-6.62627939019852	-0.12637091772701
C	-5.19205163396503	-3.16307532567737	-0.50525398006475
H	-3.28418247728487	-3.20916971118367	-1.47642113200130
C	-4.76903026730337	-0.35185188692499	-0.27759923666223
C	-3.27749200533967	1.72578925791889	-0.14770031494021

C	-6.20521330655516	-2.34200953479692	0.05009994883169
N	-6.55432856391059	-5.16528017770323	-0.06932112238063
C	-4.54658886689536	1.09684883084432	-0.08517758301675
C	-3.19911858621205	3.13874284902628	-0.20217436040003
C	-6.02365849120543	-0.92398421507266	0.12102869061330
C	-7.60359668124512	-4.40382035289188	0.46108394858220
C	-7.41147487364332	-2.93149152565840	0.50866301748393
C	-5.70315813175485	1.92035642983271	0.15327589062480
C	-8.07797922267135	2.20277951182378	0.64862400275721
C	-4.31979830735328	3.94028024586776	-0.10766885321963
C	-6.98495384364069	1.35050140587373	0.45477377045572
C	-7.11600244132639	-0.10932016089519	0.55539579915447
C	-5.58548420829533	3.34521513740421	0.11589531264890
C	-7.95515067545188	3.59528471486030	0.56583711245194
C	-4.16856576382448	5.41740313374863	-0.17949748568446
C	-8.42788085024934	-2.12436045916577	1.00136635024314
C	-6.71966738454087	4.17284642659470	0.30798177819038
C	-8.28537065316426	-0.72998128769016	1.00824285249965
O	-8.62452958693947	-4.94100301701060	0.86133872035192
H	-2.22423194249274	3.62098598287310	-0.29468604430239
O	-3.09244985502660	5.95224435937160	-0.39312060306033
H	-9.06178616367299	1.78569946712014	0.86247805400624
C	-6.60311936849761	5.65240701644000	0.23944502155213
C	-5.19440662708716	7.64717597261818	-0.05570777498883
N	-5.32520783607534	6.18161431667032	0.01336078653335
H	-8.82279954841204	4.24153087159794	0.71039925087739
O	-7.56786436110280	6.38824880324555	0.37271942083585
H	-9.34694811232205	-2.59155930012997	1.35997744860916
H	-2.90938314809172	-0.78206530344752	-1.28947520528438
C	2.14814591775827	-1.02468577067715	0.35289679673380
C	1.70026044409925	-0.21717400189785	-0.67065215306217
C	0.36825659003941	0.22825465471374	-0.44661407886790
C	-0.20489841981179	-0.24424408847767	0.73814313415884
H	2.30887815633588	0.03489697856452	-1.53955480791146
C	-1.53664024347079	0.20233102097915	0.96267358476621
C	-1.98375166517245	1.01086679960573	-0.06026878603325
H	-2.14522145412845	-0.04915379427191	1.83177717085252
S	0.91758229670724	-1.25753334288198	1.60075419620965
S	-0.75410274769550	1.24152230169363	-1.30939370533876
H	-9.11891940162844	-0.13001656113675	1.37284990123737
C	5.11057975411258	-8.32539524838707	-0.89027781967447
H	4.59349315038386	-7.85326498542505	1.16456635058233
H	6.34901077989922	-8.01477144662238	0.86441503302414
C	5.00197490063506	-9.84429174354619	-0.75017271940481
H	4.19058006333563	-10.12743884447294	-0.05837298132602
H	5.93790313757362	-10.28093217743755	-0.36200102692731
H	4.79230986229973	-10.31796816783689	-1.72215753282241
H	5.91369701976683	-8.07017861409209	-1.60205512876899

H	4.17172283202353	-7.91771337296217	-1.30173668417899
C	6.35921536642498	7.34779884063058	-0.83062161218457
H	7.96728931995189	6.81084579302569	0.52648447460548
H	6.36659124435524	6.97438312520435	1.30808640089885
C	6.55003156994974	8.86181381173879	-0.73721039226328
H	6.02160797890045	9.28103142423052	0.13564859059868
H	6.16035925162186	9.36694919421770	-1.63532552120786
H	7.61611927508858	9.12853034766341	-0.64113629181817
H	5.28856060028488	7.11101388769713	-0.95062188178462
H	6.87885160828326	6.95713809707407	-1.72176979491301
C	-5.37532786744861	8.20097039594170	-1.47209880499339
H	-4.19575372317792	7.89858357964121	0.32439348333288
H	-5.95141757057983	8.06975142246282	0.61783205771307
C	-5.22969580991201	9.72224441300343	-1.51575293743940
H	-5.36345103817367	10.10398218566193	-2.54024540899868
H	-4.23198812251036	10.04170131446498	-1.16950203985673
H	-5.97846778471668	10.21759644624568	-0.87503361013903
H	-6.37082358272917	7.90730262981098	-1.84559895789644
H	-4.62949583118360	7.73316789298996	-2.13684211655704
C	-7.37194571058972	-7.10616980070123	-1.43274939742280
H	-7.35869034634802	-6.90727139847110	0.72926151189022
H	-5.73644358282231	-7.07539903304861	-0.00543797232373
C	-7.53983259696002	-8.62557560780415	-1.46333900853301
H	-8.18283754688502	-8.97644555206614	-0.63818026357729
H	-6.56843255890572	-9.14008970133414	-1.37182976153175
H	-8.00137515820348	-8.95482224439970	-2.40794176070000
H	-6.74395831125518	-6.78030555208117	-2.27897173579685
H	-8.35350718528560	-6.61649503167175	-1.55052084491240

Table S12: Optimized geometry of **TT2**.
120
Coordinates from ORCA-job SM_7_gr_opt

C	8.68029017937708	-0.48309313102788	-0.08711903795088
C	8.20240794520764	0.82713295056782	-0.09342665099930
C	6.82494578375731	1.11124282029398	-0.11462575926528
C	5.90500097122113	0.01722387725256	-0.13001883660679
C	6.40455153517771	-1.31915691180365	-0.12579273467269
C	7.79238622822418	-1.56023701008423	-0.10378155714968
C	5.49275474394396	-2.42027349792739	-0.13959386787485
C	4.13680519064367	-2.20104176131749	-0.15719460289294
C	3.60439296637938	-0.87917909355110	-0.16265413133224
C	4.48871718176738	0.23645734821460	-0.14946917333273
C	3.97127002716734	1.57403900574113	-0.15487027943470
C	6.29980924086411	2.47980008086310	-0.12126916519111
C	4.88340051387823	2.68068741263296	-0.14232728846667
C	2.56540058994447	1.80982755315274	-0.17181082264265
C	2.09090166653891	3.15179511567913	-0.17962260211540
C	2.95606319585196	4.22100952145946	-0.17107538399880

C	4.36720536856514	4.01013504437824	-0.15149029504393
C	7.13649521393730	3.60980597982695	-0.10733949788392
C	6.61994196538635	4.90573313631365	-0.11483172260609
C	5.24086260593376	5.11631573889106	-0.13776918919156
C	2.39848699120004	5.60097497851635	-0.18407462518448
C	4.71127688366146	6.50386943019813	-0.14859313193685
C	5.99981400958113	-3.82062121450595	-0.13447149685536
C	8.32408057745051	-2.94730340293650	-0.09770374493238
N	3.31751150834574	6.65575245100502	-0.19165458185606
N	7.38831239841491	-3.99151084455089	-0.13246544693975
C	7.90512190284001	-5.37122709809597	-0.12998641533569
O	1.19681654178355	5.81521739834991	-0.19063078448393
O	5.44368293674346	7.47979911328061	-0.12452759442875
O	5.24916305048561	-4.78252197571509	-0.13396797918038
O	9.52080082423124	-3.18404821585406	-0.06574572851385
C	2.78017948581067	8.02748673455104	-0.21215953176238
H	9.75296155075283	-0.68550050661821	-0.06933474676267
H	3.47340524269417	-3.06874243789102	-0.16615149134866
H	8.92897546717940	1.63944485823537	-0.08084173187126
H	8.22002667007989	3.48858693116601	-0.09007783917353
H	1.02365410295979	3.37503761177307	-0.19391857867935
H	7.28446796716765	5.77151170144317	-0.10340474415304
C	-2.14362883810048	-3.17459978211099	-0.21877667467212
O	-1.24954998799982	-5.83806218800016	-0.23154346800370
C	-2.45120811391131	-5.62375036069517	-0.23889390911522
C	-2.61816082858106	-1.83264010099524	-0.20955292921132
C	-3.00885013779195	-4.24377990371324	-0.22601311521298
H	-1.07628794292791	-3.39785077820626	-0.22120764350263
C	-4.02413287788206	-1.59682903482727	-0.20708449874741
C	-3.65718435245292	0.85636639862721	-0.19553402700458
C	-4.42012583950950	-4.03286891106707	-0.22228346278934
N	-3.37010618958151	-6.67837266427939	-0.26348826934350
C	-4.54160045066452	-0.25926245031792	-0.19925880922280
C	-4.18959582190772	2.17822284402987	-0.18694926693619
C	-4.93636751131667	-2.70345251152176	-0.21154057839984
C	-4.76429957884136	-6.52653435384318	-0.23773435011521
C	-5.29392206380883	-5.13901335667565	-0.22551813801676
C	-5.95801057696832	-0.04003449906172	-0.19422553590588
C	-8.25570312227463	-0.84997072954121	-0.18941392602417
C	-5.54565640938237	2.39744586986114	-0.18267271768159
C	-6.87808772266611	-1.13404438246100	-0.19657481129608
C	-6.35292551995551	-2.50257029055396	-0.20591103478087
C	-6.45756107040496	1.29633194485361	-0.18657969415671
C	-8.73361827971009	0.46022014879185	-0.17964058443191
C	-6.05274283988105	3.79777196510372	-0.17321038601327
C	-6.67317279002821	-4.92845157937472	-0.21816417675343
C	-7.84555650793820	1.53736069621739	-0.17860243535575
C	-7.18975093203912	-3.63255098657789	-0.20906261383535

O	-5.49703532518164	-7.50247296496927	-0.22859118453786
H	-3.52613930954858	3.04592175205466	-0.18256622331372
O	-5.30217902046727	4.75959699108700	-0.15721110546050
H	-8.98236758359467	-1.66229086116002	-0.19088745097934
C	-8.37728965599938	2.92439116554429	-0.16881435426468
C	-7.95807223516241	5.34842421232199	-0.17909390875576
N	-7.44117717573686	3.96874762721878	-0.18565067051137
H	-9.80641694945750	0.66263991392716	-0.17295515330619
O	-9.57429103497348	3.16097768298078	-0.14873180554644
H	-7.33780280093646	-5.79422373346771	-0.22014058302712
C	2.20422556785079	-0.62194546216132	-0.17819790303828
C	1.67306886006392	0.68324945317554	-0.18099279458599
C	-2.25694074316786	0.59911361687856	-0.19779654889853
C	-1.72577379778759	-0.70607822309072	-0.20280097054460
C	-0.29022056717293	-0.65505319511047	-0.19772140694427
C	0.23755231841164	0.63222591283329	-0.19115276127605
S	-1.02095061151822	1.84428558232980	-0.19073630720324
S	0.96827612894620	-1.86711754911681	-0.19155896870329
C	-8.14979459086888	5.91293308632100	1.23230217319465
H	-8.91503358803001	5.33439257283795	-0.71606496521467
H	-7.24144702372801	5.96290569193381	-0.73966210078029
C	-8.68922864527520	7.34412194024982	1.20681039760043
H	-8.82008599787128	7.73669527116129	2.22759163668406
H	-9.66834822217712	7.39416025247132	0.70158701196070
H	-8.00318868080251	8.02314133106316	0.67278437364270
H	-7.18325885829666	5.88492043011662	1.76256832275536
H	-8.84373917661383	5.25987657581838	1.78828185040513
C	2.53253232629773	8.59961434384019	1.18724295322022
H	1.84156541213744	7.99511153540508	-0.78017773157605
H	3.50561674284685	8.64864037063152	-0.75356244314666
C	1.96721366031817	10.02005523814283	1.13172805079229
H	2.65730631301888	10.70784704727848	0.61425681726724
H	1.79587210641403	10.41815224942911	2.14430932026753
H	1.00416546783421	10.04723399236252	0.59486437795757
H	3.48179300686920	8.59431854498754	1.74865097959548
H	1.83311375890646	7.93828012283538	1.72629954339571
C	8.08144048691297	-5.94565709638353	1.27940231752732
H	8.86781005202156	-5.35367670755972	-0.65653521818886
H	7.19454789864321	-5.98164190563681	-0.70256189649557
C	8.62017529505567	-7.37701211465668	1.24960230898144
H	8.73990184763657	-7.77685865327141	2.26893422783147
H	9.60459935098238	-7.42420029239019	0.75449780374359
H	7.93943056995443	-8.05173527137759	0.70350173254852
H	8.76983581276916	-5.29697535371671	1.84722729723710
H	7.10932502989228	-5.92064886779212	1.79955889941912
C	-2.83287407366522	-8.05015087118021	-0.28352283880055
C	-2.60546445851909	-8.62948976836505	1.11637691004490
H	-1.88637564974505	-8.01544136421061	-0.83813306454700

H	-3.55085305970960	-8.66821038500894	-0.83825066971858
C	-2.04073487713962	-10.05019439683378	1.06176280881164
H	-1.07024139770296	-10.07559589645752	0.53840288176516
H	-2.72405615042994	-10.73468873649678	0.53112202364079
H	-1.88406346837665	-10.45352429274850	2.07466140738119
H	-3.56258948905486	-8.62600885079845	1.66427156746248
H	-1.91317804636381	-7.97150132872823	1.66854034527986
H	-8.27341066733140	-3.51133106011634	-0.20430946246900

Table S13: Optimized geometry of **Ph1** anion.
124
Coordinates from ORCA-job SM_2_gr_opt_ani_opt

C	5.61940909447442	-5.69799568895344	-0.28144179212706
C	6.25200702271741	-4.46155162086820	-0.37280534383436
C	5.55830113712376	-3.25223424304595	-0.16531605155503
C	4.15860453055398	-3.29893330299172	0.14704671074097
C	3.51663802864482	-4.57943492455755	0.20990163851368
C	4.25529319266753	-5.77138383403907	0.00818099945888
C	2.12459045447606	-4.65228391827217	0.46891931474778
C	1.38547731568254	-3.47960487173925	0.55962585425037
C	1.97349758818803	-2.20445323881999	0.46500255495224
C	3.39708073557809	-2.09921107191376	0.38243465797175
C	4.14236631955140	-0.85008738185563	0.56894781441818
C	6.22828206852331	-1.95872273628222	-0.25557514905641
C	5.52574181037682	-0.79251688048930	0.18350635722327
C	3.58211789060513	0.29689195906806	1.17028054067525
C	4.29299360896217	1.48897346875517	1.29496584385669
C	5.59981143298059	1.59687123696713	0.81625593605232
C	6.23376173244549	0.45343104161805	0.26744325220019
C	7.55609581908373	-1.82372867661235	-0.70681906967413
C	8.21297788983062	-0.59513536854184	-0.68595630331461
C	7.57685185642721	0.54127933009400	-0.18316203712049
C	6.31467550057636	2.88218745440317	0.91802160529827
C	8.31214421209463	1.81928689817485	-0.12047813685543
C	1.43777248896816	-5.95479987965380	0.57281640051724
C	3.60061105409956	-7.09107354372776	0.10281803475225
N	7.63786093787389	2.91026430809608	0.44615574416883
N	2.23057165531782	-7.10073079493475	0.40376136864799
C	1.56732884797642	-8.40809551636184	0.51118189882867
O	5.80458344056672	3.89252170727380	1.38863726627997
O	9.46064062797504	1.94610896405347	-0.53212746531527
O	0.23735436981237	-6.05863378879442	0.79514103248965
O	4.20548176477820	-8.14446435669006	-0.06423669147121
C	8.36145922448828	4.18647721064371	0.53039277567294
H	6.17462074100308	-6.62515595005266	-0.43451728346406
H	0.30203904015016	-3.56781000815214	0.66166822578110
H	7.31809013306592	-4.44149942005847	-0.59809117823931
H	8.09507858140769	-2.69363282528668	-1.08158727587516

H	3.83330681676965	2.36207151553797	1.76235560615393
H	2.56800236248600	0.26222851440701	1.55961284297457
H	9.24089202163342	-0.50498641334331	-1.04180278082219
C	-4.18007666482331	-1.54478084120982	-0.95540762864369
O	-5.69952789269476	-3.94553112759305	-1.03293653434415
C	-6.19168353861880	-2.94037065292350	-0.53301063790190
C	-3.46807357100787	-0.35188419579465	-0.84576410362634
C	-8.20824495769186	-4.25997402588899	-0.05300554601042
C	-5.47693900482374	-1.65371630928568	-0.45011911782176
H	-3.72857701102049	-2.41818754890328	-1.43013145774528
C	-4.01918825803189	0.79596627193722	-0.23756715062097
C	-1.84971426620574	2.15145820055266	-0.16806489657380
C	-6.10224045427668	-0.50967176669622	0.10742537785824
N	-7.49180055716718	-2.97821188179895	-0.00147636560542
C	-3.27224163015785	2.04602460934250	-0.06644770279268
C	-1.26466219191052	3.42652262134099	-0.28154777695308
C	-5.39590533607589	0.73837678157809	0.17081112050583
C	-8.17086995552141	-1.87700373361464	0.53949722534790
C	-7.43788225295500	-0.59689684226081	0.57985586532218
C	-4.03146611356683	3.24617837522744	0.17298642425369
C	-6.11919517147313	4.40987411096204	0.71276950657387
C	-2.00371313533903	4.59905619410883	-0.18713324511218
C	-5.42664679607091	3.19987766237986	0.50503824848353
C	-6.09317000705070	1.90611848259081	0.61407158291446
C	-3.39124764827885	4.52668787295271	0.09531141080651
C	-5.48955481319842	5.64631699655405	0.60303594932710
C	-1.32116740182312	5.90171684818885	-0.31421202996657
C	-8.06875335684243	0.54221966884356	1.08345462707956
C	-4.12884937209725	5.71920455488611	0.29704237919088
C	-7.41369537063343	1.77191942895880	1.08667329913011
O	-9.31981840028298	-1.99795297710074	0.95174314516989
H	-0.18313049342332	3.51537033328171	-0.40130382668467
O	-0.13060556910126	6.00609865155506	-0.58463229666905
H	-7.18219591084488	4.39015295560712	0.95212503729167
C	-3.47750169770947	7.03906732978338	0.18381802769158
C	-1.44305529762874	8.35411961063776	-0.23008011213623
N	-2.10478858456021	7.04748495535107	-0.10611979783361
H	-6.04423161550775	6.57399767193096	0.75484273691834
O	-4.08722522864853	8.09284644772109	0.32939718494677
H	-9.09115577402929	0.45285701243020	1.45498499870228
H	-2.46071846675888	-0.31686797918422	-1.25258543980980
C	1.02310561071161	-1.06516701137377	0.33086778519398
C	-0.05792515920256	-0.90357876934782	1.22154966495235
C	1.11775059030584	-0.16683294066251	-0.74979342439144
C	0.18244060148294	0.85105847394469	-0.92540686048127
C	-0.89848569523584	1.01303276845287	-0.03452959667684
C	-0.99254863049257	0.11488734122583	1.04650387142092
H	1.93296196418145	-0.27599153934407	-1.46916943121745

H	-1.80722900639483	0.22440308641158	1.76636094787721
H	-0.15150816498188	-1.57156928157496	2.08176341617107
H	0.27583601737400	1.51831637996479	-1.78619716217834
C	1.03009548098848	-8.92306235555520	-0.82854816032986
H	0.74324839097199	-8.29320770947750	1.22765087368375
H	2.30575833021123	-9.11279779934297	0.91662662808703
C	0.34683171994982	-10.28441926065261	-0.68822658110398
H	-0.03316606671980	-10.64121085562841	-1.65880268747197
H	-0.50883766518114	-10.23489626443945	0.00671723615998
H	1.04568009242791	-11.04697876622635	-0.30345152242883
H	1.86702611789691	-8.99422260052534	-1.54407144574403
H	0.31700328994162	-8.18497689561714	-1.23335616383268
C	8.19465198018136	5.05768279387699	-0.71970168828312
H	9.42233157985370	3.94819531029589	0.68391363420775
H	7.97767820979831	4.71690363206805	1.41188732556516
C	8.95265881294765	6.38090892255561	-0.60510780556609
H	10.03490400024903	6.21389692129531	-0.46800235253054
H	8.59602716859414	6.97528969317197	0.25382035729035
H	8.82149405376997	6.99400134991527	-1.51169991745222
H	7.12032023564806	5.25211580104408	-0.87729085630636
H	8.55455221519142	4.49383312935110	-1.59715020294692
C	-1.46096056117644	8.90357006513186	-1.66069511055660
H	-0.40716909595264	8.22631350975955	0.11087030726533
H	-1.96392425144362	9.04457989964618	0.44628743021663
C	-0.77419811321257	10.26640330179448	-1.76249694982710
H	0.28139594421735	10.20774053357878	-1.44745721807839
H	-1.27095250616075	11.01591010845954	-1.12305600584182
H	-0.79240418032561	10.64601411393350	-2.79702362951468
H	-2.50787972157820	8.98414775162810	-1.99913158409936
H	-0.95984041835912	8.18048231144565	-2.32583316947893
C	-9.01456762500231	-4.45006585389479	-1.34266769494052
H	-8.87855573041166	-4.28984284689970	0.81651886383808
H	-7.45570340172117	-5.05412167482945	0.04119626371063
C	-9.75313771446300	-5.78885953572839	-1.36719089712591
H	-10.45972088933720	-5.87471066249116	-0.52367684567094
H	-9.05027548390457	-6.63684413724024	-1.29993140325939
H	-10.33021982297289	-5.90900069894924	-2.29832909760186
H	-8.32845524759408	-4.38384336436868	-2.20416752306282
H	-9.73688526890562	-3.62145491516910	-1.43711624800634
H	-7.94817156824883	2.64331142088475	1.46444843180136

Table S14: Optimized geometry of **Ph2a** anion.

120

Coordinates from ORCA-job SM_5_gr_opt_ani_opt

C	7.77082311817788	-1.46831875611321	0.16350335475303
C	7.17480678381289	-0.26633307565056	0.52703816787243
C	5.77109818031972	-0.13908620142448	0.64027646603326
C	4.95918690240597	-1.28035812504540	0.34798965656985

C	5.58419251708665	-2.51860733832889	0.00390937253808
C	6.99236485608751	-2.60522304894965	-0.09005203756584
C	4.77757617154315	-3.66533534116331	-0.24195619451104
C	3.39520643804463	-3.58234465329391	-0.15310790769159
C	2.73884490112237	-2.37143688719665	0.15924027932318
C	3.52740774308241	-1.21020848310373	0.40548866637618
C	2.89235949286788	0.01792796951002	0.76853011031525
C	5.12566060747080	1.09316671840312	1.07583553630898
C	3.69930567199632	1.13897818065116	1.15779755444600
C	1.46768764675909	0.11905232960348	0.76884710740770
C	0.88142569435585	1.27275711299259	1.35719516764978
C	1.64359592111775	2.34295216578007	1.78510287492573
C	3.06399461685560	2.32043757859104	1.64782961644315
C	5.85888933067538	2.24652112292133	1.44446434300439
C	5.22930919975219	3.39310314532957	1.90878824760069
C	3.83168401084143	3.43896120554829	2.02934140205622
C	0.97039620954739	3.51259469335186	2.38774642105750
C	3.18124954982132	4.64793237929832	2.57151983385219
C	5.40281913340295	-4.95537758661677	-0.59548314462749
C	7.64504460154522	-3.87671404931356	-0.44842008201522
N	1.78896651815296	4.59652821626222	2.74440172107424
N	6.80461412160077	-4.98274820806731	-0.65934249866539
C	7.44163645648297	-6.26127787696396	-1.00534693329018
O	-0.23964290482833	3.56427545145924	2.56896606474978
O	3.80772266669961	5.65532762418974	2.88087864908389
O	4.75282793878630	-5.97008822367556	-0.82424982968948
O	8.86112832027880	-3.99339587786772	-0.55994609589681
C	1.13167122172279	5.77757021836108	3.32251949008116
H	8.85649810281275	-1.54993596113554	0.07912232764901
H	2.83132905051531	-4.49124461630854	-0.36077837083716
H	7.81734625408158	0.58948994219854	0.73374467992515
H	6.94708572092216	2.24316752393967	1.37788854541349
H	-0.19442658644621	1.33282924851391	1.50936121221759
H	5.80854637373889	4.27132297454332	2.20020939766068
C	-3.84902467913706	-3.01643729210416	0.54256871003487
O	-5.55415914421169	-5.15018153497141	1.27876100896408
C	-6.03881279905474	-4.04748353363842	1.04668507784605
C	-3.01747592409373	-1.92994006636405	0.19264316734437
C	-8.24345895652922	-4.99396474433067	1.56838399281881
C	-5.22630932197259	-2.88155044172670	0.64656225915359
H	-3.42899001304800	-3.99535503324316	0.77045143399362
C	-3.62032481687908	-0.66829089682901	-0.08255745022174
C	-1.38452527024344	0.31845286960925	-0.49174340826084
C	-5.84911265086318	-1.63102999781705	0.37372344560922
N	-7.42309870781350	-3.84579537899318	1.15813191322999
C	-2.80767721407221	0.43726060808508	-0.48337699207830
C	-0.63254017447895	1.35139018740336	-1.11425373493853
C	-5.04502984635278	-0.51530438601578	-0.01496704515163

C	-8.08962027986420	-2.64045015108904	0.88061572846227
C	-7.25285705035531	-1.49687658554723	0.47741483909357
C	-3.43600804718393	1.65801004163367	-0.90103968330107
C	-5.40273010096900	3.07468152110102	-1.21364685544614
C	-1.22424301210990	2.51598312075666	-1.56523071886586
C	-4.85185743752615	1.83353021900963	-0.81304519675939
C	-5.67448504190214	0.72841684201003	-0.33744513875615
C	-2.63018971277368	2.71560808551299	-1.42241996453445
C	-4.60855513333313	4.09723941093547	-1.71330914652302
C	-0.38330300667829	3.55537088929796	-2.19496116135499
C	-7.84979958090276	-0.26213801734824	0.19103984008776
C	-3.22068209726890	3.92709093686342	-1.83471070405093
C	-7.07983564876317	0.82238334531891	-0.21212164735374
O	-9.31042056323724	-2.57181962034036	0.97908939166445
H	0.43830163934209	1.23921430520794	-1.27295324246530
O	0.81472044012893	3.40930062379694	-2.40261209571329
H	-6.47793412950679	3.24120674566659	-1.14399535817664
C	-2.39664615716960	5.00858596231209	-2.40792669141064
C	-0.19130043576892	5.81926420327699	-3.12618604927407
N	-1.02161396397242	4.75761949770090	-2.54024008192904
H	-5.04884535292888	5.04454612892598	-2.02985010119636
O	-2.86828014987328	6.08094782242103	-2.76946321930045
H	-8.93419721525555	-0.17241056556862	0.28376825397593
C	1.28370714717100	-2.27086054606976	0.19263733110481
C	0.66451258598952	-0.99535403970755	0.29400371675523
C	-1.56422822502332	-2.05390610443272	0.15324105243928
C	-0.91724391768782	-3.31652561281406	0.22863258667805
C	0.45219662329260	-3.42226968090121	0.15198007275847
C	-0.75819687912404	-0.89099721121038	0.01473275724407
H	0.90150421741551	-4.41499425554482	0.12454110108111
H	-1.51095254267689	-4.22901010745292	0.28607459631080
H	-7.58552089625983	1.76074572977782	-0.43955995565667
C	7.62011633163676	-6.45734978872880	-2.51421265119044
H	6.80927608195840	-7.05930348014949	-0.59392481622188
H	8.41821331600200	-6.27968695507902	-0.50435788380334
C	8.27818188491495	-7.79748490008980	-2.84615917994007
H	8.39997531857859	-7.92393320682572	-3.93393814122108
H	7.67408973472136	-8.64402560738140	-2.47732786800828
H	9.27786619711199	-7.87905698178208	-2.38715874824041
H	8.23198942306279	-5.62964637879572	-2.91097158597162
H	6.63267383754334	-6.39396078451583	-3.00136744234832
C	-8.70805769969976	-5.85645055360312	0.39027364568785
H	-9.11263887544812	-4.59178444845517	2.10476698839621
H	-7.63714589889068	-5.59625027959922	2.25800227628771
C	-9.56620201335727	-7.03788537002567	0.84496766092141
H	-9.00969979237934	-7.69692864523242	1.53349239323294
H	-9.88902753757175	-7.64887072919261	-0.01316786353095
H	-10.47349359343569	-6.69665757755959	1.37175064484678

H	-7.82125228197336	-6.22303774272321	-0.15377826677754
H	-9.27992434759300	-5.22377142889787	-0.30990544323171
C	0.66738877571609	6.78648681806946	2.26727242502650
H	1.85395878752985	6.24872294482833	4.00234435848735
H	0.27107721238272	5.41707848487478	3.90152620379239
C	-0.08189415889044	5.72965884779097	-4.65184914944561
H	0.80497874056386	5.73721804741862	-2.67217886836094
H	-0.64170451713067	6.77770669483958	-2.83624945159957
C	0.78687659896750	6.84598192415114	-5.23225398327461
H	1.81206595592257	6.80899353021600	-4.82683147196585
H	0.37348557634415	7.84155461828227	-4.99547739961085
H	0.85876286527973	6.76619319331968	-6.32900789405189
H	-1.09478719282758	5.77689515564313	-5.08651788511838
H	0.34044178673623	4.74672220206036	-4.92108162534458
C	-0.01251265490758	8.00403593084511	2.89552324585695
H	0.67088082443330	8.53749430170856	3.57872906435975
H	-0.90305047583128	7.71154983125632	3.47748946694768
H	-0.33859244407958	8.71910359484987	2.12349273640457
H	-0.02819794359405	6.28256086811828	1.57482584472156
H	1.54002306851696	7.10736308657120	1.67354085875868

Table S15: Optimized geometry of **Ph2b** anion.
120
Coordinates from ORCA-job SM_6_gr_opt_ani_opt

C	7.39824377497240	-2.91636730580369	0.19542223761892
C	7.42082677611999	-1.52810184161894	0.19689227850605
C	6.23096986466613	-0.76391323034502	0.19738301274429
C	4.97613201715835	-1.45199847936092	0.19672834586224
C	4.96824074085213	-2.88188649074155	0.19726771274530
C	6.17925796044953	-3.60733089289886	0.19615278020780
C	3.72349475850014	-3.57417708416007	0.19390345065635
C	2.53342328641398	-2.86872274157138	0.19183104135663
C	2.49506400083978	-1.45541009175269	0.19391729073101
C	3.72994969101343	-0.73875899710072	0.19583946205232
C	3.73803858488710	0.69577720531727	0.19598290567704
C	6.23916974294767	0.69263293158248	0.19776497257407
C	4.99215959297261	1.39487809147533	0.19719347089768
C	2.51131603224871	1.42635441043916	0.19405032506286
C	2.56559481447973	2.83913226595637	0.19204945656440
C	3.76353478516728	3.53111349725593	0.19456800540976
C	5.00038697110622	2.82476398029314	0.19817055085549
C	7.43758107049984	1.44336438158519	0.19784404944292
C	7.43062683241866	2.83179595877537	0.19701727378076
C	6.21950202892834	3.53645336751799	0.19767562434052
C	3.74272460490586	5.00901228258004	0.19291609924676
C	6.23235893451742	5.01167288327726	0.19697479660983
C	3.68610377754481	-5.05174948493965	0.19213911680122
C	6.17556592879926	-5.08262170808992	0.19482599064082

N	4.98651928262869	5.65800196052190	0.21322958755009
N	4.92248432412002	-5.71479483482906	0.21167903668811
C	4.90289246993159	-7.18421976715823	0.21511882130638
O	2.70515180023949	5.66012661623127	0.17626626999575
O	7.26850023760850	5.66705712379358	0.18471503584634
O	2.64126007026250	-5.69117490096638	0.17620013658032
O	7.20425537900163	-5.74957553738949	0.18148225773388
C	4.98403564116581	7.12753986259829	0.21684553092933
H	8.32629586323736	-3.49125441430438	0.19404673158850
H	1.61744975708084	-3.45813124171538	0.18831244988102
H	8.38904632277288	-1.02805353179748	0.19730939802226
H	8.40022331406842	0.93255979189014	0.19806534033724
H	1.65637583974216	3.43892351242865	0.18818211212696
H	8.36505684599392	3.39625767822098	0.19616123365114
C	-2.56659080886286	-2.83960700681598	0.19233997635339
O	-2.70616574101904	-5.66059529052050	0.17676500287110
C	-3.74373868544550	-5.00948286405941	0.19331397170623
C	-2.51229813864474	-1.42681648376487	0.19430779160776
C	-4.98505516492419	-7.12800274293527	0.21739651092406
C	-3.76452821182054	-3.53156924325245	0.19496372946229
H	-1.65737332237937	-3.43940120724302	0.18839727638643
C	-3.73901095624604	-0.69623672736202	0.19633526430343
C	-2.49602894334700	1.45493667139540	0.19416595047092
C	-5.00137638885033	-2.82520737743350	0.19865059474285
N	-4.98752647782414	-5.65846253629103	0.21377013233026
C	-3.73091431339466	0.73830428875297	0.19618265047737
C	-2.53437260900476	2.86825973367652	0.19209110305843
C	-4.99313934784193	-1.39532540924665	0.19764554050469
C	-6.23335839493476	-5.01212453642169	0.19768010292005
C	-6.22049365609449	-3.53688896890889	0.19827148848429
C	-4.97709068522280	1.45155402333870	0.19715010542886
C	-7.42177941528898	1.52768439643655	0.19746521374203
C	-3.72443028828358	3.57371876675520	0.19424345533712
C	-6.23193697910943	0.76348621274520	0.19788907038891
C	-6.24014574445077	-0.69307194267484	0.19828981230926
C	-4.96918473193013	2.88143844295661	0.19768154772279
C	-7.39918587057146	2.91595941507180	0.19597813379856
C	-3.68702390228806	5.05130081301298	0.19247999227680
C	-7.43160702649605	-2.83223237326172	0.19767312762904
C	-6.18019230996791	3.60689366733675	0.19663030031703
C	-7.43855296281457	-1.44379148356971	0.19846170514317
O	-7.26950371407329	-5.66748881502578	0.18541401507390
H	-1.61839172696906	3.45765558147449	0.18851259749702
O	-2.64217017730424	5.69070307817520	0.17652478221814
H	-8.39000322432411	1.02764290038537	0.19795254467747
C	-6.17648937257900	5.08219609764902	0.19529314860845
C	-4.90377838473321	7.18378217356348	0.21551224341375
N	-4.92338925076598	5.71435760514609	0.21208778469368

H	-8.32720904831066	3.49089662480541	0.19465752506672
O	-7.20516809529850	5.74913702936504	0.18193952159957
H	-8.36604153482478	-3.39668664632813	0.19691543291606
C	1.23181838744969	-0.72496100697062	0.19373530485058
C	1.23990196717680	0.71025214267866	0.19376055700582
C	-1.23279302386765	0.72448269293816	0.19386677744881
C	-1.24088025557963	-0.71072872589315	0.19388502086116
C	-0.00832544840180	-1.38021798270161	0.19390715117103
C	0.00735230372065	1.37973949969890	0.19392218310989
H	-0.01493507429538	-2.46648947379896	0.19457141459609
H	0.01396331659153	2.46601043302120	0.19460528568680
C	4.89184902885741	-7.78913049127573	-1.19258343983472
H	4.00402797924024	-7.49338681993293	0.76476456871289
H	5.79466418608664	-7.51770805715758	0.76201994648794
C	4.86819797742003	-9.31756870138027	-1.16220118591161
H	4.86029916908458	-9.73708911823625	-2.18067647206657
H	3.97281073524874	-9.69568818982184	-0.63961840966730
H	5.75214005937311	-9.72402331622099	-0.64119367310912
H	5.78398893713225	-7.43748470225498	-1.73879045333092
H	4.00923167620583	-7.41050504905170	-1.73575809788536
C	-4.98731411223362	-7.73311573171507	-1.19026322958972
H	-5.87832423164098	-7.45098111944431	0.76821066104828
H	-4.08751633277957	-7.44756845171971	0.76317403602532
C	-4.98422988708470	-9.26173062527195	-1.15979815878498
H	-5.87218171076697	-9.65601580623589	-0.63624503419889
H	-4.09263514781305	-9.65204157902589	-0.63971843108693
H	-4.98500759363116	-9.68138366684127	-2.17824863668261
H	-4.10169021221769	-7.36650683681853	-1.73690309989337
H	-5.87665433952249	-7.36944641472978	-1.73303617576220
C	-4.89272312255021	7.78867673636174	-1.19219680829314
H	-4.00491158808665	7.49294613006779	0.76515725209659
H	-5.79554655580075	7.51728642966119	0.76240898248102
C	-4.86906531575455	9.31711584536655	-1.16183722936764
H	-3.97369889546365	9.69523853181368	-0.63922051233784
H	-5.75302905571385	9.72358893388197	-0.64088204073653
H	-4.86111658077677	9.73661727208809	-2.18032092958389
H	-5.78486024603383	7.43702775066517	-1.73840717599845
H	-4.01010336184712	7.41003955154887	-1.73535875637021
C	4.98598408582487	7.73265469272152	-1.19081447953827
H	5.87742179506350	7.45052632122268	0.76746455319224
H	4.08661196145549	7.44710156673384	0.76281651467284
C	4.98293013360808	9.26126884751512	-1.16033411460467
H	4.09147909318835	9.65158677731415	-0.64001093295924
H	4.98345300805616	9.68094215180396	-2.17877518785007
H	5.87102364976585	9.65553277056578	-0.63700571714787
H	4.10023117295080	7.36605956095509	-1.73725325659879
H	5.87519660144216	7.36897869453289	-1.73379207375346
H	-8.40119324710292	-0.93298255878191	0.19874016441287

REFERENCES

1. S. Stoll and A. Schweiger, *Journal of Magnetic Resonance*, 2006, **178**, 42-55.
2. T. Liu and A. Troisi, *Adv. Mater.*, 2013, **25**, 1038-1041.
